# Questionnaire-based analysis of autism spectrum disorders and gastrointestinal symptoms in children and adolescents: a systematic review and meta-analysis

**DOI:** 10.3389/fped.2023.1120728

**Published:** 2023-07-26

**Authors:** Huizhong Gan, Yanhong Su, Linlin Zhang, Guolin Huang, Ciai Lai, Ying Lv, Yongchun Li

**Affiliations:** ^1^ Department 2nd Clinical Medical College, Organization Guangzhou University of Chinese Medicine, Guangzhou, China; ^2^ Department Nanfang Hospital, Organization Southern Medical University, Guangzhou, China

**Keywords:** autism spectrum disorder, gastrointestinal, children and adolescent, prevalence rate, questionnaire

## Abstract

**Background:**

Gastrointestinal (GI) symptoms are frequently experienced by children with autism spectrum disorder (ASD), and these symptoms cause difficulties for these children and their families. However, studies of GI symptom prevalence differ significantly. This meta-analysis aimed to analyze the prevalence of GI symptoms in children with ASD.

**Methods and findings:**

PubMed, Scopus, Web of Science, EMBASE were electronically searched to collect all literature on gastrointestinal symptoms of children with ASD collected through questionnaires or scales from January 2012 to May 2021. Four researchers independently scanned the literature and extracted information on general characteristics. First author name, year of publication, geographical location, type of study, sample sizes of ASD and control (if any) children, sex and average age, number of GI cases, number of GI symptoms, GI assessment tools (gastrointestinal symptoms scale), autism diagnosis methods, and other necessary data were collected and analyzed using Stata V16. The questionnaires included the Rome, 6-GSI, GIQ, GSRS, GSIQ, ADI-R, PedsQL-GI, parent-report, GI-related, and self-administered questionnaires. Compared with typically developing (TD) children, the odds ratio for In children with ASD with at least one GI symptom was 3.64, and the total prevalence was 55%. The cumulative prevalence rates of various symptoms were summarized, showing that 37% of children with ASD had constipation, 21% had abdominal pain, 19% had diarrhea, 8% had vomiting, and 23% had abdominal distension.

**Conclusions:**

The results of this meta-analysis on GI symptoms in ASD show that patients with ASD are more likely to develop symptoms than TD children. The prevalence of GI symptoms in In children with ASD was 55%.

**Systematic Review Registration:**

www.crd.york.ac.uk/PROSPERO, identifier, #CRD42017080579.

## 1. Introduction

Autism spectrum disorder (ASD) is a neurodevelopmental disorder characterized by communication or social difficulties, persistent and circumscribed repetitive behavior, interest, or activity patterns that limit or impair daily function; it is characterized by symptoms in early childhood (1). The prevalence of autism spectrum disorder has shown an upward trend. In the United States in 2018, the overall prevalence rate of ASD in men and women was 3.4 per 1,000 whereas; early identification of 8-year-old children indicated an overall prevalence of 23.0 per 1,000 (2–4).

Children with ASD are often reported to have gastrointestinal (GI) symptoms and other complications (5). GI symptoms in individuals with ASD can include, GI symptoms can include constipation, diarrhea, nausea, abnormal defecation, abdominal pain, and vomiting. Although GI symptoms are not clearly identified as core symptoms or comorbidity of autism, associated GI dysfunction is often mentioned (6–8). GI dysfunction has also been related to sleep disorders and food allergy, which lead to other metabolic disorders, physical and intellectual disability, and endocrine dysfunction. These, in turn, may lead to higher risks of overweight or obesity in children (5, 7, 9). GI symptoms play a critical role in complications found in children with ASD. Ames and coworkers showed that adolescents with autism had more complex needs for psychiatric and health care, which meant they had to pay more money or effort for interventions (10); children with ASD and gastrointestinal symptoms paid more money or effort for interventions. Children and adolescents with autism have a higher rate of hospitalization for gastrointestinal disorders than the general population (11). In 2014, McElhanon et al. reported that children with ASD have higher prevalence of GI symptoms than TD children, through a study investigating gastrointestinal symptoms in In children with ASD. However, this article did not calculate the specific prevalence of GI in children with ASD. In recent years, with the increase of related research reports, the results may change and need to be further updated (12).

Since then, several reviews have explored the GI profile of ASD. One review in 2018 of the ASD-related gastrointestinal literature by Holingue et al. found a prevalence of at least one GI symptom ranging from 4.2% to 96.8% (median 46.8%) (13). A 2021 systematic review of prevalence and comorbidities in children with ASD noted a significant difference in the prevalence of GI symptoms, ranging from 0.00 to 67.80 (5). No new meta-analysis has been conducted since 2014.

At present, the collection of data and investigation of GI symptoms in children with ASD come mostly from parents’ reports by questionnaires, medical records, or physician's diagnosis. One study has shown that the ratio of agreement between physician diagnosis and parental report for GI symptoms in children with ASD is 92.1% (14). Compared with other sources, questionnaire surveys are widely used for data collection in health services research and can be administered through the mail, and face-to-face or telephone interviews. They have the advantages of being cost-effective and easy to administer on a large scale.

The current research on GI symptoms in children with ASD is insufficient. The prevalence of GI symptoms in In children with ASD varies greatly among different studies. There are different assessment methods for symptoms in these children, and reliable and effective GI evaluation tools for ASD research are lacking. As studies are updated, more attention is being paid to GI problems in children with ASD, and this provides an opportunity for better assessments. The purpose of this meta-analysis was to integrate studies on the GI conditions of children with ASD that have been collected using questionnaires or scales, most of which were cross-sectional studies, to determine the relationship between ASD and the prevalence of GI symptoms. To integrate the prevalence of ASD combined with GI, and update the meta-analysis of GI in ASD after 2014.

## 2. Materials and methods

### 2.1. Search strategy

This systematic evaluation and meta-analysis followed the recommendations of the Preferred Report Project (PRISMA) reporting guidelines for systematic evaluation and meta-analysis. The protocol for this study was registered in PROSPERO (#CRD42017080579, www.crd.york.ac.uk/PROSPERO).

We used a four-pronged, systematic approach to identify relevant publications. We included PubMed, Scopus, Web of Science, and EMBASE with a combination of the following keywords and Medical Subject Headings (if applicable) and we limited the search to title, summary, and keywords. The retrieval formula was (stomach OR intestines OR abdomen OR gastrointestinal OR alimentary OR belly OR digestive) AND (infantile autism) OR autism OR (autism spectrum disorder) OR (child autism) OR (Kanner syndrome) OR (Asperger syndrome). The search was further limited by the period from January 1, 2012, to October 1, 2021, to keep the focus on recent research.

### 2.2. Inclusion and exclusion criteria

Rayyan (https://www.rayyan.ai/) was used to screen the retrieved literature by title and abstract in a collaborative online manner, enabling blinding to ensure that researchers were screened independently; the blinding was removed once all the studies were screened. Differences in opinion between researchers about study eligibility were resolved through discussion and finally adjudicated by the lead researcher (HZG), as required.

#### 2.2.1. Inclusion criteria

We included children and adolescents (age <18 years) diagnosed with autism. Participants were diagnosed with ASD according to the Diagnostic and Statistical Manual of Mental Disorders (DSM) or the International Classification of Diseases (ICD) classification and/or based on structured and valid diagnostic tools [the Autism Diagnostic Observation Sheet (ADOS) and/or the Autism Diagnostic Interview (ADI)]. The method used in the literature was questionnaire assessment of gastrointestinal symptoms. There are specific symptom frequency statistics.

#### 2.2.2. Exclusion criteria

In addition to articles that do not qualify for inclusion, we will exclude animal models or other model studies, systematic reviews or meta-analyses, letters, notes and conference abstracts, and studies published in non-peer-reviewed journals. Articles with lack of data regarding specific symptoms, were also excluded.

## 3. Data extraction

We extracted the following information from each study: first author's name, year of publication, geographical location, study type, sample sizes of children with ASD and controls (if any), sex and mean age, number of GI cases, number of GI symptoms, GI assessment tool (Gastrointestinal Symptom Scale), and autism diagnosis modality. Three investigators (HZG, YHS, GLH) retrieved, selected the literature, and did the data extraction from the literature independently. For continuous outcomes, the data were pooled as mean (*M*) or Standard Deviation (SD) with 95% confidence intervals (95% CI) and for dichotomous outcomes, as a odds ratio (OR) with 95% CI.

## 4. Quality assessment

Three researchers (YHS, GLH, LLZ) independently assessed the quality of the included studies and selected appropriate scales according to the types and characteristics of the included literature. Disagreements among review researchers regarding the risk of bias in particular studies was to be resolved through discussion and ultimately adjudicated by the lead researcher (HZG).

MINORS (15) was used in clinical trials, NOS (16) was used in observational studies including cohort and case-control studies, AHRQ (17) was used in cross-sectional studies, and ROB2 was used in RCTS.

## 5. Statistical analysis

Data from qualified studies were used in the meta-analysis. The prevalence of GI in ASD was pooled by single-group rate meta-analysis. The odds ratio (OR) was used as an effect analysis statistic for the counting data. Point estimates and 95% CI of each effect are given. Heterogeneity among the studies was measured by the heterogeneity index *I*^2^ Assessment. *I*^2^ ≤ 50% indicated that there was no obvious heterogeneity, and the fixed effect model was adopted; *I*^2^ > 50% indicated that there was heterogeneity. The source of heterogeneity was determined by gradually removing a single study; if *I*^2^ > 50% remained, the random effect model was used. The results of the meta-analysis are represented by forest maps. Subgroup analysis was conducted to explore the potential heterogeneity of the publication region, publication year, study type, and questionnaire type. Funnel plotting and Egger's test were used to assess for publication bias; *p* < 0.01 was considered sufficient evidence of no publication bias. When the influence deviation of a study on the overall results was *p* > 0.05, that study was excluded. Documents with missing data were excluded from both the summary and the itemized analysis. Stata V16 was used for statistical analysis, and a *p* value <0.05 was considered significant.

## 6. Results

### 6.1. Search results

**Figure 1:**
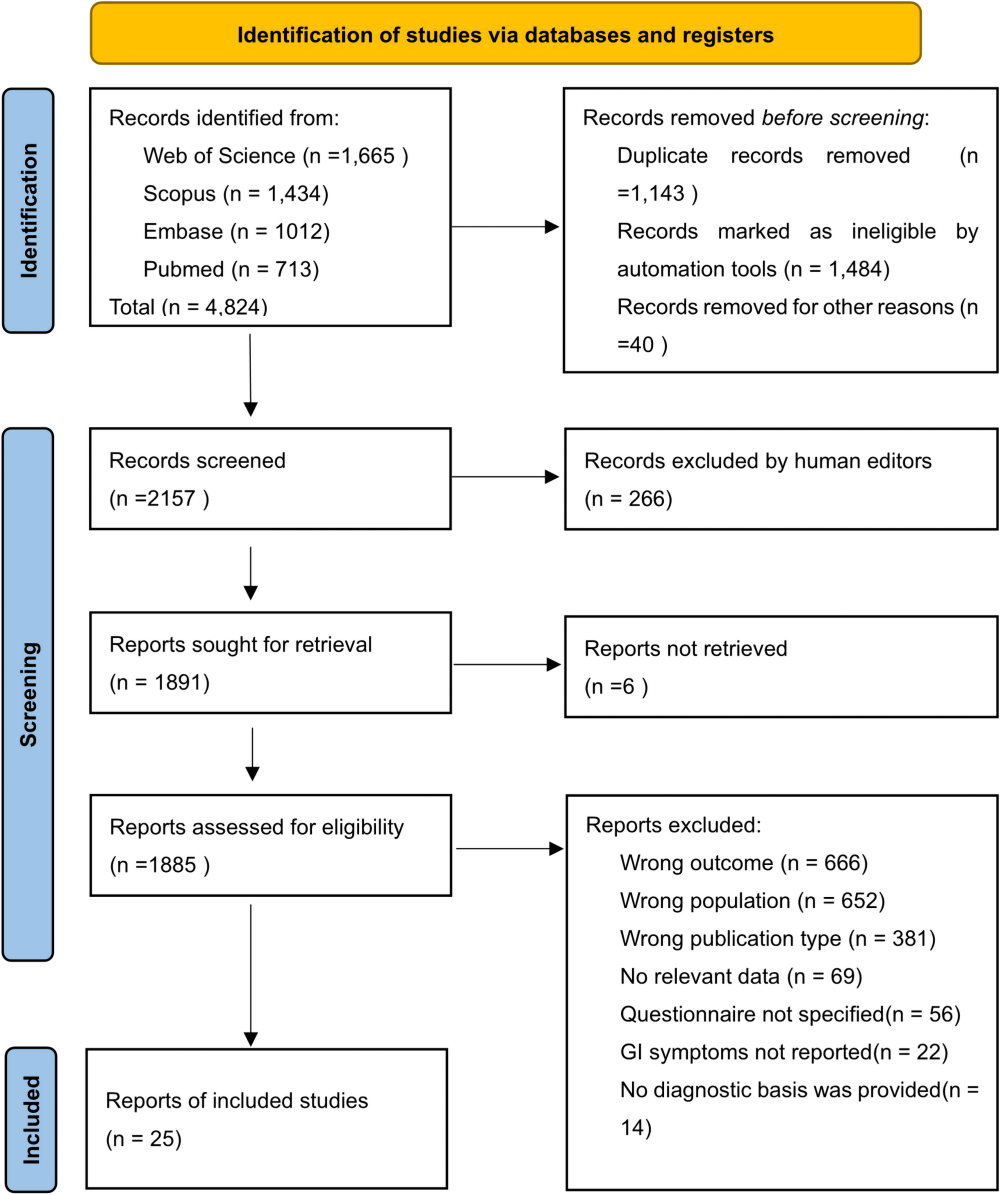
PRISMA flow chart of study selection in this review. *From:* Page MJ, McKenzie JE, Bossuyt PM, Boutron I, Hoffmann TC, Mulrow CD, et al. The PRISMA 2020 statement: an updated guideline for reporting systematic reviews. *BMJ* 2021;372:n71. doi: 10.1136/bmj.n71.

A total of 4,824 studies were retrieved from the four databases, with 2,157 studies remaining after re-checking. A manual re-check yielded 1,891 studies. Ultimately, 25 studies (18–41) were screened (Figure 1). Nine were from Asia, five from Europe, nine from the Americas, and two from Africa. The articles included cross-sectional studies (17), clinical trials (3), cohort studies (2), case-control studies (2), and randomized control trials (1). The questionnaires included the Rome, 6-GSI, GIQ, GSRS, GSIQ, ADI-R, PedsQL-GI, parent-report, GI-related, and self-administered questionnaires (see Table 1 for details). A total of 4,723 children with ASD were included, ranging in age from 0.5 to 18 years. The mean and standard deviation of age were calculated in 18 articles, and the overall mean [standard deviation, SD] age was 5.82 [3.09] years. Twenty articles provided the sex differential of study subjects; 81.9% were male. We included 16 medium-quality, one low-quality, and eight high-quality studies (Table 2).

**Table 1:** The scale information of included articles.

Author	Year	*N*	Constipation	Abdominal Pain	Diarrhea	Nausea	Bloating	Aerophagia	ARS	PDS	IBS	Stool Smell	Loose Stools	Pain on Stooling	GRD	Blood in Stool	Blood in Vomit	APBS	Stomach Discomfort	LAC	NFI	Excessive	Assessment of GI
Reynolds et al. (18)	2020	672	+	+	+	+	−	+	−	−	−	−	+	+	−	−	−	−	−	+	−	−	GIQ
Restrepo et al. (19)	2020	255	+	+	+	+	+	−	−	−	−	−	−	+	−	+	+	−	−	−	−	−	The Gastrointestinal History (CHARGE GH) questionnaire
Cheng et al. (20)	2020	323	+	+	+	+	+	−	−	−	−	+	−	−	−	−	−	−	−	−	−	−	QPGS-RIV
Ferguson et al. (21)	2019	120	+	+	−	−	−	−	−	−	+	−	−	−	−	−	−	−	−	−	−	−	QPGS-RIII
Li et al. (22)	2020	94	+	+	+	+	−	−	−	−	−	+	+	−	−	−	−	−	−	−	−	−	The self-complied general condition questionnaire
Kang et al. (23)	2017	18	+	+	−	+	−	−	−	+	−	−	+	−	+	−	−	−	−	−	−	−	GSRS、DSR
Fulceri et al. (24)	2015	115	+	+	+	+	−	−	−	−	−	−	−	+	−	−	−	−	−	−	−	−	CBCL 1½–5
Leader et al. (25)	2021	129	+	+	+	+	+	−	−	−	−	−	−	−	−	−	−	−	−	−	−	−	Questionnaires for Pediatric FGID
Leader et al. (26)	2020	136	+	+	+	+	+	−	−	−	−	−	−	−	−	−	−	−	−	−	−	−	The GI Symptom Inventory (Autism Treatment Network 2005)
Son et al. (27)	2015	59	+	+	+	+	+	−	−	−	+	+	−	−	−	−	−	−	−	−	−	+	ROME III
Margolis et al. (28)	2018	131	+	+	+	−	−	−	−	−	−	−	−	−	+	−	−	−	−	−	−	−	The 35-item Gastrointestinal Symptom Inventory
Lai et al. (29)	2020	107	+	+	+	+	−	+	+	+	+	−	−	−	−	−	−	−	−	−	+	−	QPGS-III
Prosperi et al. (30)	2017	163	+	+	+	+	−	−	−	−	−	−	−	+	−	−	−	−	−	−	−	−	CBCL 1½–5
Sanctuary et al. (31)	2019	8	+	+	+	+	+	−	−	−	+	−	+	+	−	+	+	−	−	−	−	−	GIH survey 、 QPGS-RIII
Bresnahan et al. (32)	2015	195	+	−	+	−	−	−	−	−	−	−	−	−	−	−	−	−	−	−	−	−	18- and 36-month questionnaires
Khalil et al. (33)	2021	58	+	+	+	−	+	−	−	−	−	+	+	−	−	−	−	−	−	−	−	−	6-GSI questionnaire
Wong et al. (34)	2021	69	+	+	+	+	−	+	+	+	+	−	−	−	−	−	−	−	−	−	−	−	Rome IV Diagnostic Questionnaires for Pediatric FGID
Chakraborty et al. (35)	2021	176	+	+	+	+	+	+	−	−	−	−	−	−	+	+	−	−	+	−	−	−	PedsQL-GI
Ghodsi and Kheirouri (36)	2019	36	+	+	−	+	−	−	+	−	−	−	−	−	−	−	−	−	−	−	+	−	ROME III
Shaaban et al. (37)	2017	30	+	+	+	−	+	−	−	−	−	+	+	−	−	−	−	−	−	−	−	−	6-GSI
Gok et al. (38)	2020	102	+	+	+	+	+	+	+	−	−	−	−	−	+	+	−	−	−	−	−	−	parent reports a questionnaire
Kumar et al. (39)	2021	52	+	+	+	+	−	−	+	−	−	−	−	−	−	+	−	+	−	−	−	−	Rome IV
Fields et al. (40)	2021	1,244	+	−	+	+	−	−	−	−	−	−	+	+	−	−	−	−	−	−	−	−	parent-administered health-history forms
Liu et al. (41)	2016	154	+	−	+	+	−	−	−	−	−	−	−	−	−	−	−	−	−	−	−	−	The mealtime behavioral questionnaire
Zhu et al. (42)	2017	328	+	+	+	+	+	−	−	−	−	+	−	−	−	−	−	−	−	−	−	−	Rome IV

ARS, adolescent rumination syndrome; PDS, postprandial distress syndrome; IBS, irritable bowel syndrome; GRD, gastroesophageal reflux disease; APBS, abdominal pain with bowel symptoms; LAC, loose alternating with constipation; NRFI, non retentive fecal inconvenience; GIQ, gastrointestinal questionnaire; 6-GSI, 6-the GI severity index; PedsQL-GI, pediatric quality of life inventory-gastrointestinal symptoms scales; GIH, CHARGE gastrointestinal history survey; QPGS-RIII, the questionnaire on pediatric gastrointestinal symptoms—rome III version; CBCL, the child behavior checklist; GSRS, the gastrointestinal symptom rating scale; DSR, the daily stool record.

**Table 2:** Study characteristics in the systematic review and meta-analysis.

Author	Year	Country	Study design	Number of participants	Age (yr)	Gender (M/F)	Evaluation method	Score	Diagnosis of autism
Reynolds et al. (18)	2020	USA	Cross-sectional	A: 656	A: 4.63 ± 0.57	A: /	AHRQ	(7/11)	ADOS, ADI-R
				DD = 938, POP = 851	DD: 4.65 ± 0.63; POP: 4.63 ± 0.64	DD: 626/312; POP: 459/392			
Restrepo et al. (18)	2020	USA	Cross-sectional	A: 255	A: 3.02 ± 0.47	A: 184/71	AHRQ	(5/11)	ADOS, ADI-R
				T: 129	T: 2.93 ± 0.56	T: 75/54			
Cheng et al. (19)	2020	China	Cross-sectional	A: 323	A: 4.72 ± 1.33	A: 274/49	AHRQ	(7/11)	DSM-V
				T: 180	T: 4.74 ± 0.93	T: 142/38			
Ferguson et al. (20)	2019	USA	Cross-sectional	A: 120	A: 11.8 ± 3.8	A: 108/12	AHRQ	(7/11)	DSM-IV-TR ADOS
				T: /	T: /	T: /			
Li et al. (21)	2020	China	Cross-sectional	A: 94	A: 4 ± 1.15	A: 78/16	AHRQ	(4/11)	DSM-V
				T: 90	T: 4.28 ± 0.89	T: 73/17			
Kang et al. (22)	2017	USA	Clinical Trial	A: 18	A: 10.8 ± 1.6	A: 16/2	MINORS	(19/24)	ADI-R
				T: 20	T: 11.4 ± 2.5	T: 18/2			
Fulceri et al. (23)	2015	Italy	Cross-sectional	A: 115	A: 3.8 ± 1.1	A: 95/20	AHRQ	(4/11)	DSM-IV-TR, DSM-V
				T: 115	T: 3.9 ± 1.0	T: 95/20			
Leader et al. (24, 25)	2021	Ireland	Case- control	A: 129	9.3 ± 3.84	A: 100/29	NOS	(5/9)	DSM-IV-TR
				T: /	T: /	T: /			
	2020	Ireland	Cross-sectional	A: 136	8.36 ± 4.13	A: 98/38	AHRQ	(4/11)	DSM-IV-TR
				T: /	T: /	T: /			
Son et al. (26)	2015	USA	Cross-sectional	A: 59	A: 10.3 ± 1.8	A: 52/7	AHRQ	(6/11)	ADOS
				T: 44	T: 10.0 ± 1.8	T: 21/23			
Margolis et al. (27)	2018	USA	Cross-sectional	A: 131	A: 7.8 ± 3.8	A: 108/23	AHRQ	(8/11)	DSM-IV-TR
				T: /	T: /	T: /			
Lai et al. (28)	2020	Hong Kong	Cross-sectional	A: 107	A: 9.2 ± 3.3	A: 92/15	AHRQ	(6/11)	DSM-V
				T: 249	T: 9.8 ± 3.5	T: 219/30			
Prosperi et al. (29)	2017	Italy	Cross-sectional	A: 163	A: 3.6 ± 1.15	A: 137/26	AHRQ	(8/11)	DSM-V
				T: /	T: /	T: /			
Sanctuary et al. (30)	2019	USA	RCT	A: 8	A: 6.8 ± 2.4	A: 7/1	ROB2	Hight	ADOS
				T: /	T: /	T: /			
Bresnahan et al. (31)	2015	USA	Cohort	A: 195	A: 6–36 months	A: 158/37	NOS	(8/9)	DSM-IV-TR
				DD: 4,636, TD: 40,295	DD/TD: 6–36 months	DD: 3,490/1,146; TD: 19,201/21,094			
Khalil et al. (32)	2021	Egypt	Clinical Trial	A: 58	A: 5.41 ± 1.55	A: 39/19	MINORS	(18/24)	DSM-V
				Siblings: 45; unrelated control: 45	Siblings: 4.31 ± 3.23; unrelated control: 5.36 ± 2.6	Siblings: 22/23; unrelated control: 28/17			
Wong et al. (33)	2021	China	Case- control	A: 69	A: 8.59 ± 1.58	A: 69/0	NOS	(6/9)	DSM-V
				T: 69	T: 8.35 ± 1.89	T: 69/0			
Chakraborty et al. (34)	2021	USA	Cross-sectional	A: 176	A: 5.41 ± 1.63	A: 140/36	AHRQ	(5/11)	DSM-V
				T: /	T: /	T: /			
Ghodsi et al. (35)	2019	Iran	Cross-sectional	A: 36	A: 8.64 ± 2.79	A: 27/9	AHRQ	(2/11)	DSM-V
				T: 18	T: 7.5 ± 2.61	T: 14/4			
Shaaban et al. (36)	2017	Egypt	Clinical Trial	A: 30	A: 7.06 ± 1.36	A: 19/11	MINORS	(18/24)	DSM-V
				T: /	T: /	T: /			
Gok et al. (37)	2020	Turkey	Cross-sectional	A: 61	A: 9.5 ± 3.9	A: /	AHRQ	(7/11)	DSM-IV-TR, ICD-10
				T: /	T: /	T: /			
Kumar et al. (38)	2021	India	Cross-sectional	A with GI: 20	GI: 11.5 ± 5.4;	A: /	AHRQ	(4/11)	ISAA
				No GI: 38	No GI: 12.3 ± 4.5	No GI: /			
Fields et al. (39)	2021	USA	Cross-sectional	A: 1,244	A: 2–5 years	A: 1,020/224	AHRQ	(8/11)	SCQ, ADOS、ADI-R
				DDS: 1,244; POP: 1,487	DD/POP: 2–5 years	DD: 836/408; POP: 791/696			
Liu et al. (40)	2016	China	Cross-sectional	A: 154	A: 5.21 ± 1.83	A: 141/13	AHRQ	(6/11)	DSM-V
				T;73	T: 4.83 ± 0.84	T: 67/6			
Zhu et al. (41)	2017	China	Cross-sectional	A: 328	A: 5.1 ± 1.6	A: 285/43	AHRQ	(7/11)	DSM-V
				T: 202	T: 4.9 ± 0.8	T: 106/96			

yr, year; A, autism spectrum disorder; T, typical development; PDD-NOS, pervasive developmental disorder not otherwise specified; DD, developmental delays; POP, the general population; DSM, the diagnostic and statistical manual of mental disorders; ADOS, the autism diagnostic observation schedule; ADI-R, the autism diagnostic interview-revised; ISAA, Indian scale for assessment of autism; SCQ, the social communication questionnaire.

### 6.2. Publication bias

This study explored the prevalence of GI symptoms in children with ASD. Therefore, the publication bias test was performed on 11 studies (Table 3) (18–27) that recorded GI symptoms in the ASD and typically developing (TD) groups and 19 studies (Table 4) (18–35) that collected the total number of GI cases in children with ASD.

**Table 3:** Egger's test of 11 studies recording the prevalence of GI cases in ASD and TD.

Std_eff	Coef.	Std. err.	*t*	*p *>* *|*t*|	[95% conf. interval]
Slope	1.31446	0.1875111	7.01	0.000	0.8902807, 1.73864
Bias	−0.0293982	0.9568174	−0.03	0.976	−2.19387, 2.135073

**Table 4:** Egger's test of 19 studies recording the single group prevalence of GI cases in ASD.

Std_eff	Coef.	Std. err.	*t*	*p *>* *|*t*|	[95% conf. interval]
Slope	0.3973801	0.0985541	4.03	0.001	0.1894491, 0.6053112
Bias	1.74281	1.489766	1.17	0.258	−1.400321, 4.885941

The *p* values of the Egger test were greater than 0.05, indicating no publication bias.

### 6.3. Study questionnaires

Different questionnaires were used in the included studies, which can be roughly divided into the Rome, 6-GSI, Gastrointestinal symptoms, parent-report, GI section of the child behavior checklist (CBCL), and self-made questionnaires. Among these, the Rome IV questionnaire was used in four studies and the Rome III questionnaire in another five. The 6-GSI was selected by two studies; the CBCL was used in two. One study used a self-made questionnaire, and two were parent-report questionnaires. There were eight gastrointestinal symptom questionnaires including the SEED self-built Gastrointestinal questionnaire, the Gastrointestinal System Rating Scale (GSRS), a daily stool record, the 35-item Gastrointestinal symptom checklist developed by the autism treatment network (ATN), and the Pediatric Quality of Life Inventory Gastrointestinal Symptoms Module (PEDSQL-GI). There were 21 symptoms mentioned in all studies, and the symptoms of constipation, abdominal pain, diarrhea, nausea, and vomiting were the GI symptoms included in most of the questionnaires.

**Figure 2:**
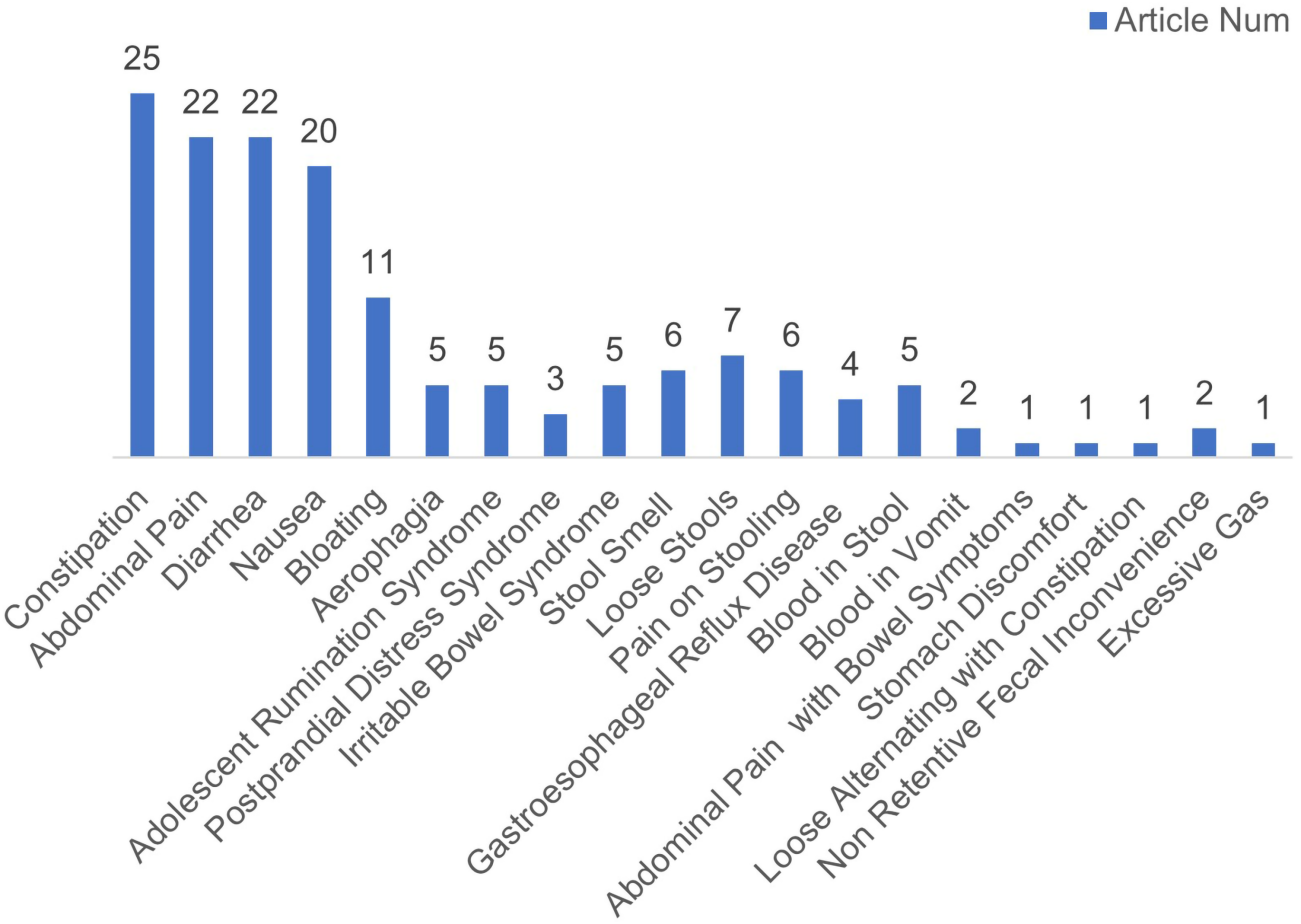
Frequency of occurrence of each GI symptom in 25 articles.

Constipation was mentioned in all questionnaires. Abdominal pain was recorded in all except for the parent-administered health history forms, the self-made 18- and 36-month forms, and the mealtime behavioral questionnaires. Diarrhea was not recorded in the Gastrointestinal Symptom Rating Scale and the daily stool records (DSRS + DSR) questionnaire or in two studies using the Rome 3 questionnaire. The self-made 18- and 36-month questionnaires, the 35-item Gastrointestinal Symptom Inventory, and the 6-GSI did not record nausea and vomiting. The collection of other symptoms is scattered (see Table 1, Figure 2).

### 6.4. Children with ASD are more likely to have gastrointestinal symptoms than TD children

**Figure 3:**
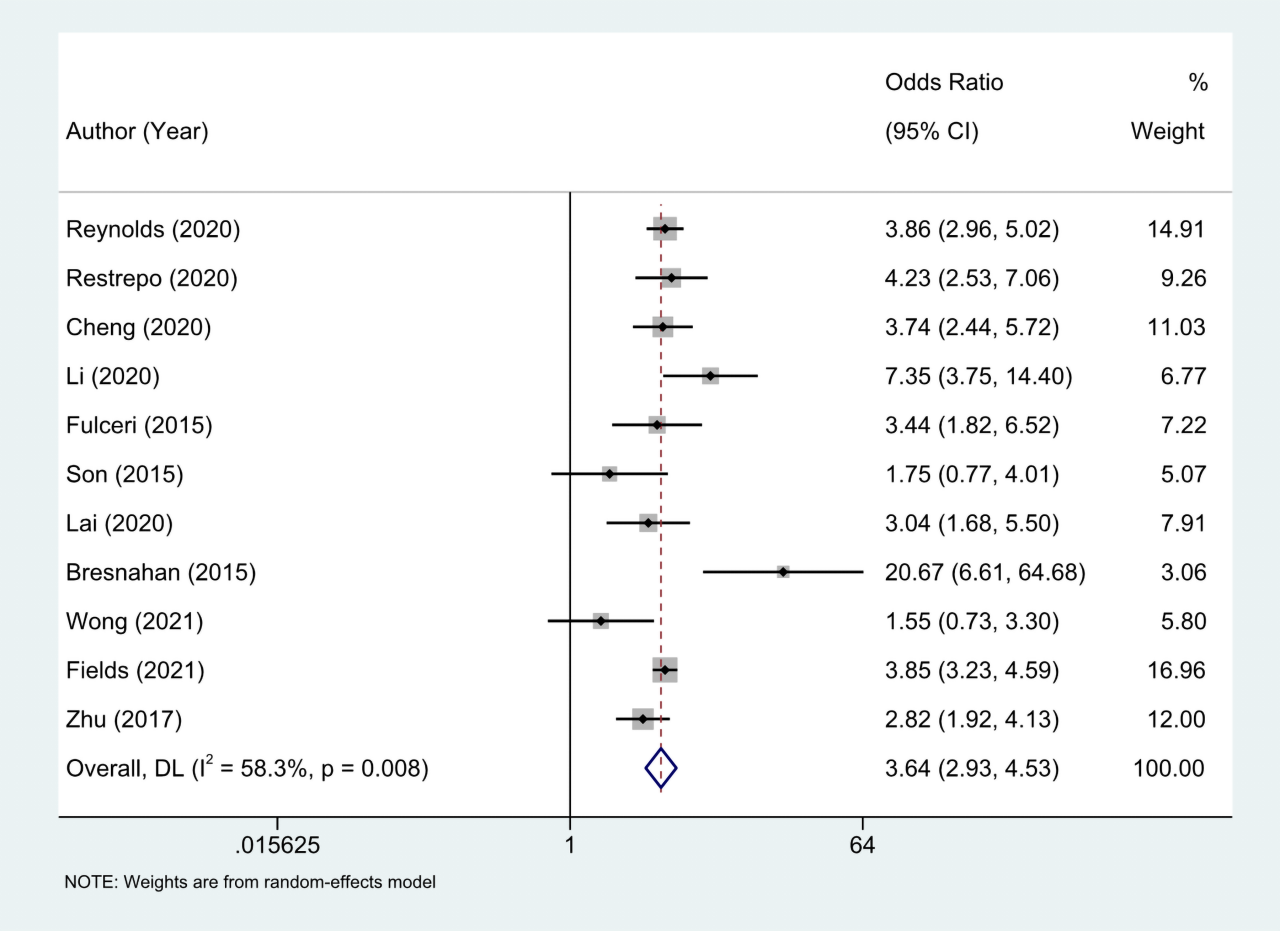
Eleven studies provided the case numbers of gastrointestinal symptoms in ASD versus TD children, OR = 3.64 (2.93, 4.53), Medium heterogeneity. Random effects model.

**Figure 4:**
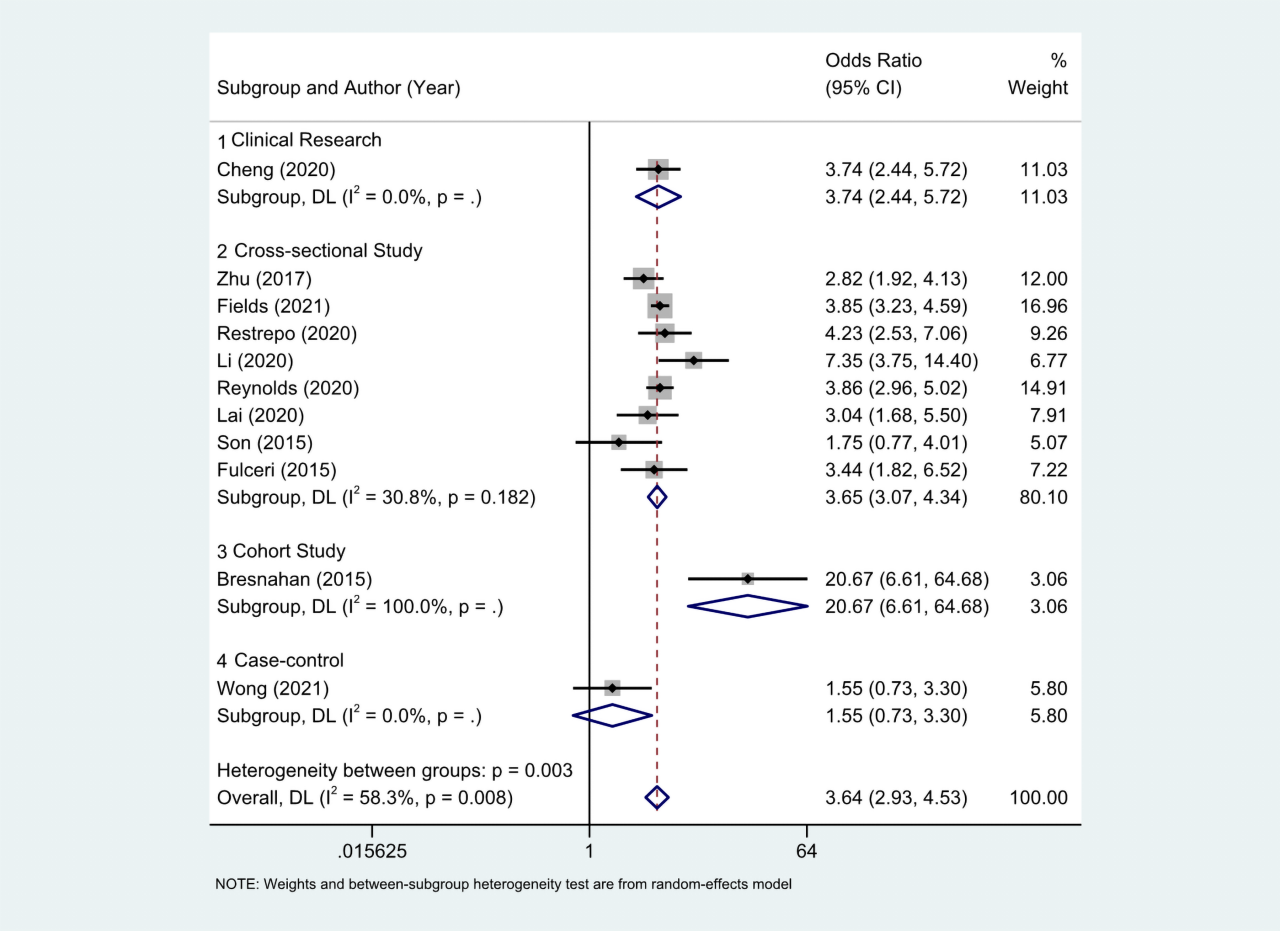
With subgroup analysis according to study type. (1) Clinical research; (2) cross-sectional study; (3) cohort study; (4) case-control.There were 8 cross-sectional studies, *I*^2^ = 30.8%, *p* = 0.182. Other research types just 1 article each, and no heterogeneity statistics. The OR of clinical research, cross-sectional, cohort study, case-control literature were 3.74 (2.44, 5.72), 3.65(3.07, 4.34), 1.55 (0.73, 3.30).

**Figure 5:**
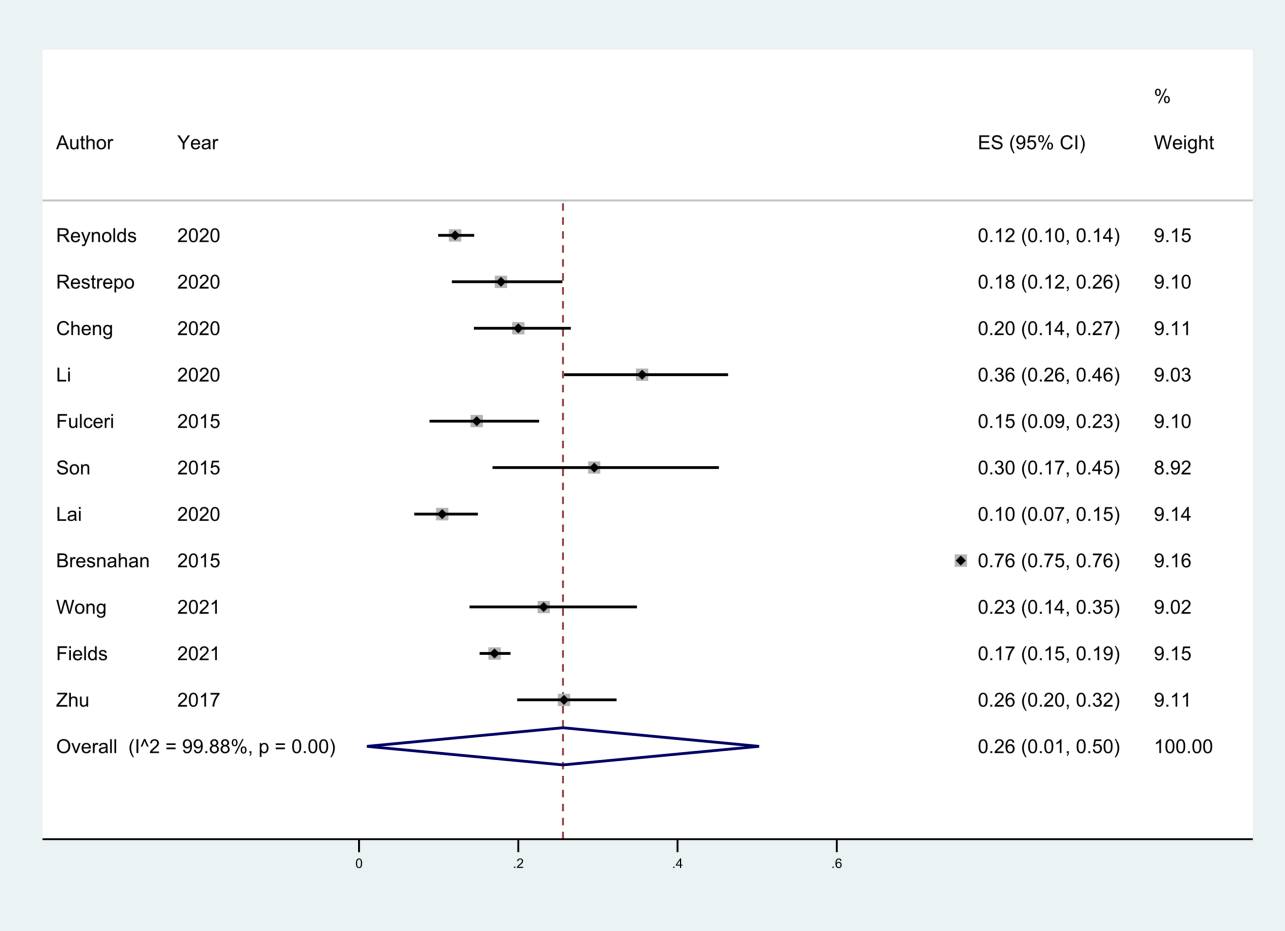
The total prevalence of GI in children in TD group was 26%, 95% CI (0.01, 0.50). Random effects model.

Eleven studies (18–27) provided the case numbers of gastrointestinal symptoms in ASD versus TD children. The meta-analysis showed that the OR of children with ASD for having GI symptoms was 3.64; that is, children with ASD were about twice as likely as TD children to have GI symptoms. *I*^2^ was 58.3% (*p* = 0.008), indicating great heterogeneity (Figure 3). With subgroup analysis (Figure 4) according to study type with *I*^2 ^= 30.8% and *p* = 0.182, heterogeneity decreased significantly, suggesting that study type may be the cause of heterogeneity, with an OR of 3.65 (95% CI: 3.07, 4.34). At the same time, our meta-analysis of the prevalence of total GI symptoms in TD children showed that the prevalence was 26%, *I*^2 ^= 99.9%, *p* < 0.05 (Figure 5). After removing the articles that differed significantly from the others, the prevalence was 19%, *I*^2 ^= 83.49%, *p* < 0.05 (Supplementary Figure S1, see the Supplementary Material).

### 6.5. GI symptoms in children with ASD compared to TD children

Eleven studies (18–19, 21, 23, 26, 28, 31, 33, 40, 41) reported constipation in children with ASD and TD, with an OR value of 2.83, *I*^2 ^= 23.6%, *p* > 0.05. Children with ASD are more likely to experience constipation than TD children (Supplementary Figure S2, see the Supplementary Material). Twelve articles (19, 21, 22, 24, 25, 32, 34–37, 39, 41) recorded cases of children with ASD complaining of simple abdominal pain. Seven studies (18, 18, 20, 23, 29, 30, 38) reported cases of defecation-related abdominal pain, and two (26, 28) found no abdominal pain. Seven studies (18–19, 21, 23, 26, 41) compared abdominal pain in ASD and corresponding TD controls, with an OR of 2.60 (95% CI: 2.07, 3.26). This finding suggests that abdominal pain is more likely to occur in ASD (*I*^2 ^= 48.7%, *p* > 0.069). However, heterogeneity in the results was low (Supplementary Figure S3, see the Supplementary Material). The prevalence of diarrhea in ASD and TD groups was reported in nine articles (18–19, 21, 23, 26, 31, 40, 41), and the combined OR was 3.27 (95% CI: 1.81, 5.91); children with ASD were more likely to have diarrhea than TD children (*I*^2 ^= 71.9%, *p* = 0.000) (Supplementary Figure S4, see the Supplementary Material). After grouping according to different research types, the OR for cross-sectional literature (18, 18, 21, 23, 26, 40, 41) integration was 3.81 (95% CI: 2.26, 6.42), *I*^2 ^= 33.3%, *p* = 0.174. Four studies (18, 19, 26, 41) compared the prevalence of abdominal distension in ASD and TD, and we calculated an OR of 1.45 (95% CI: 0.96, 2.17) (Supplementary Figure S5, see the Supplementary Material). The effect value crosses the invalid line, and the confidence interval includes 1, indicating that ASD has no influence on the occurrence of abdominal distension in children (*I*^2 ^= 21.2%, *p* > 0.05). The heterogeneity of the results was low. Combining all nine studies (18–19, 21, 23, 28, 33, 40, 41) providing data on vomiting, we obtained an OR of 3.18 (95% CI: 2.12, 4.77), suggesting that children with ASD were more than twice as likely to experience vomiting as neurotypical children (*I*^2 ^= 0.0%, *p* = 0.441) (Supplementary Figure S6, see the Supplementary Material).

### 6.6. Dietary problems in children with ASD compared to TD children

Only two studies (31, 40) mentioned food allergy in children with ASD and TD, and the OR was 1.76 (95% CI: 1.05, 2.97). Thus, children with ASD were more likely to have food allergy (*I*^2 ^= 62.6%, *p* < 0.05). One study (40) offered statistics on picky eating in children with ASD versus TD. According to the article, 41.6% of children with ASD have mild and 26% have severe picky eating. 14.5% of children with ASD have mild resistance and 9% have severe resistance to new foods. For TD children, the comparative percentages were 32.9%, 11%, 20.5%, and 1.4%, respectively. In terms of severe picky eating and resistance to new food, *p* values of the ASD and TD data were both <0.05, and thus, the differences were statistically significant. This suggests that children with ASD have more serious picky eating behaviors. In addition, the prevalence of ruminant syndrome in children with ASD has been reported as 0%–10% (28), with the prevalence in TD as 0.4%–1.5% (28, 33). The prevalence of gas swallowing in ASD was 1.5%–6.3% (20, 26, 28, 33), and in TD children was 0%–4.5% (26, 28, 33).

### 6.7. Prevalence of total GI symptoms in children with ASD

**Figure 6:**
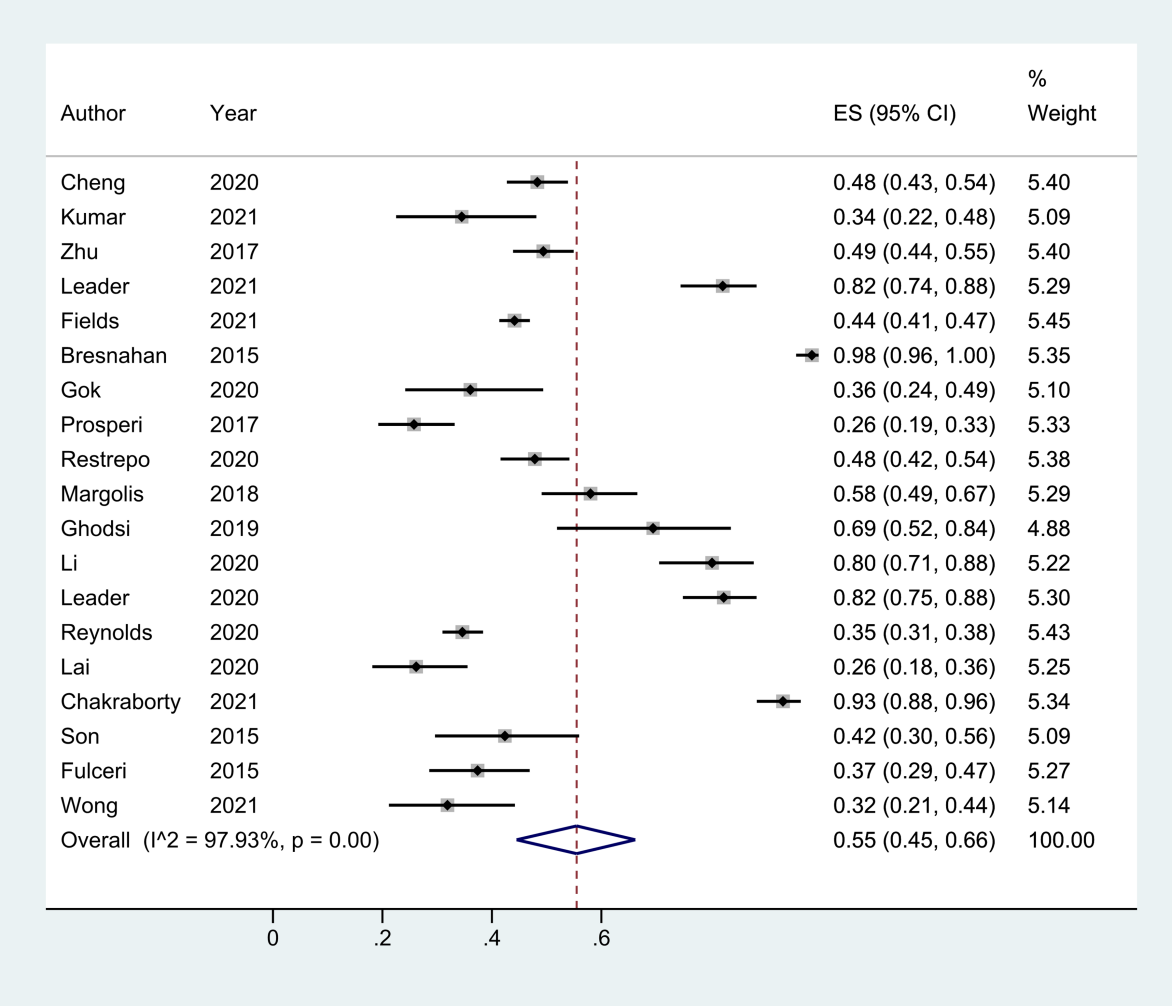
The prevalence of total GI symptoms in children with ASD based on 19 articles. It was 55% (0.45, 0.66).Random effects model.

A total of 20 studies reported the prevalence of total GI symptoms in children with ASD. One (41) of these included only ASD patients with GI symptoms and was unable to calculate the prevalence of GI disturbance, so it was excluded from the meta-analysis. Thus, data from 19 studies (22–32) were ultimately included. The cumulative prevalence was 55% (95% CI: 0.45, 0.66), and more than half of the children with ASD had at least one gastrointestinal disorder, *I*^2 ^= 97.93%, *p* < 0.05 (Figure 6).

**Figure 7:**
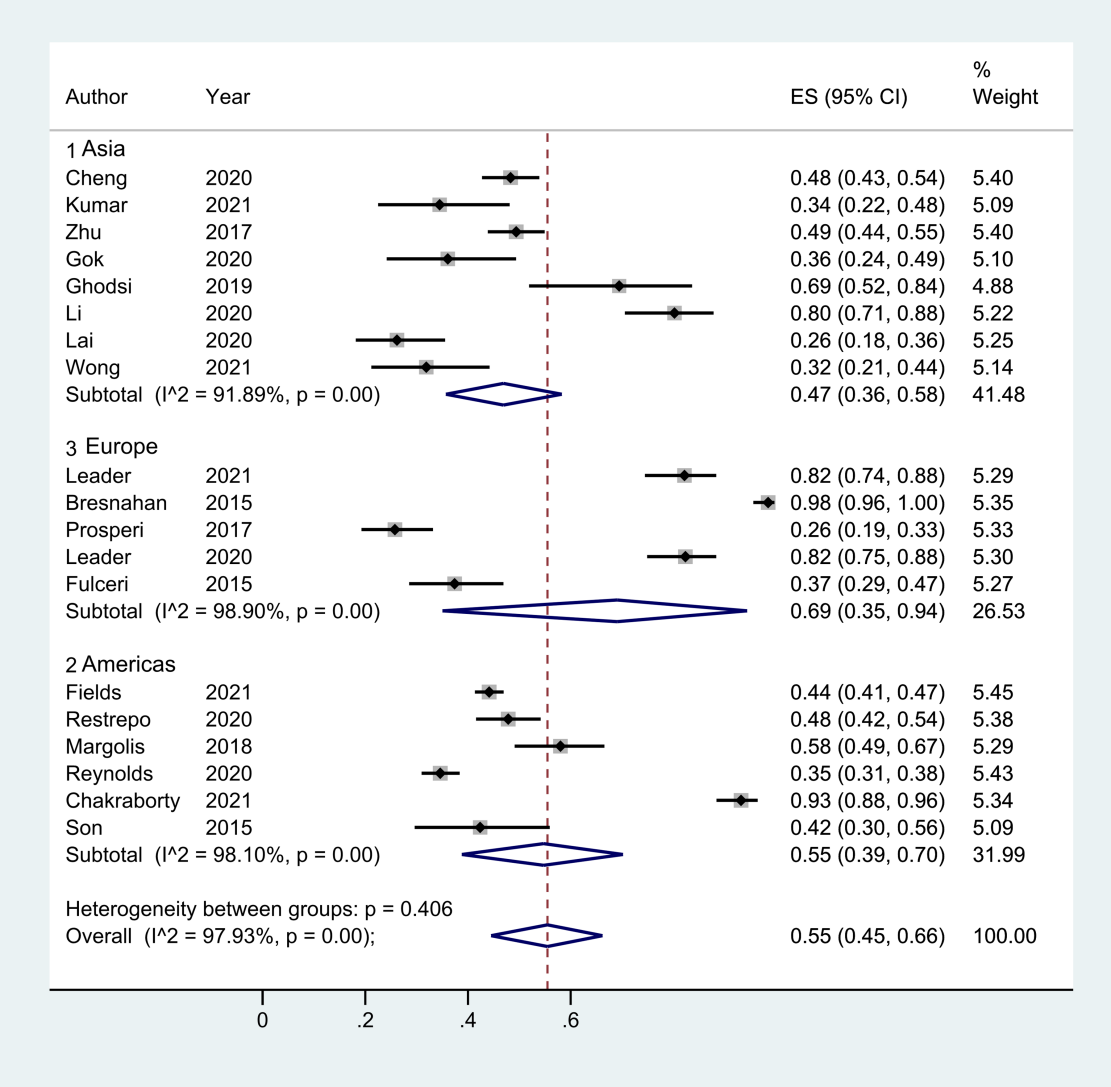
The prevalence of total GI symptoms in children with ASD grounded by region.

Meta-regression analysis based on region, year, type of questionnaire, and type of study yielded Tau2 = 0.2041 with *I*^2 ^= 96.92%; Adj R2 = −17.96%; *p* values were 0.344 (region), 0.671 (year), 0.959 (questionnaire type), and 0.922 (study type). Thus, the factors listed above were not the main sources of heterogeneity; however, the location of publication had the greatest influence on the prevalence of total GI in children with ASD. We found that the prevalence of GI in children with ASD varied when the literature was grouped by region. The prevalence was 47% (95% CI: 0.36, 0.58) in Asia, 55% (95% CI: 0.39, 0.70) in the Americas, and Europe was the highest at 69% (95% CI:0.35, 0.94) (Figure 7). The children's age, race, family income, parents’ education level, and mothers’ reproductive age could be other factors contributing to heterogeneity; however, as detailed data were not available, further analysis could not be made.

### 6.8. Prevalence of various GI symptoms in children with ASD

According to the data in the included studies, the cumulative prevalence of various symptoms showed that 37% of the children with ASD had constipation, 21% had abdominal pain, 19% had diarrhea, 8% had vomiting, and 23% had abdominal distention. All 25 articles provided information on constipation in children with ASD, with an prevalence of 37% (95% CI: 0.30, 0.43), *I*^2 ^= 96.00%, *p* = 0.00 (Supplementary Figure S7, see the Supplementary Material). A total of 22 articles (18–27, 29–32, 34–37, 39–41) documented diarrhea in children with ASD, with a combined prevalence of 19% (95% CI: 0.14, 0.24), *I*^2 ^= 96.86%, *p* = 0.00 (Supplementary Figure S8, see the Supplementary Material). Data on abdominal pain in children with ASD were reported in 21 articles (18–26, 28–30, 32, 34–37, 39, 41), with an prevalence of 21% (95% CI: 0.14, 0.28), *I*^2 ^= 96.55%, *p* = 0.00 (Supplementary Figure S9, see the Supplementary Material). Data on vomiting were collected from 17 articles (18–19, 21, 33–25, 28, 29, 33–35, 37–41), with an prevalence of 8% (95% CI: 0.05, 0.12), *I*^2 ^= 94.11%, *p* = 0.00 (Supplementary Figure S10, see the Supplementary Material). The prevalence of abdominal distension in the 12 groups (18, 19, 23–26, 30, 32, 34, 36, 41) of children with ASD was 23% (95% CI: 0.12, 0.37), *I*^2 ^= 97.63%, *p* = 0.00 (Supplementary Figure S11, see the Supplementary Material).

## 7. Discussion

This meta-analysis and systematic review collected studies on the gastrointestinal symptoms of autism published between 2012 and 2021. Updated and supplemented the meta-analysis of gastrointestinal tract in children with ASD in 2014. Using standardized data collection and rigorous inclusion criteria combined with a variety of statistical tools, the prevalence rate of gastrointestinal symptoms of autism was calculated. This review included 25 studies involving 4,720 children with ASD.

Based on the statistical analysis of the gastrointestinal questionnaires included in the studies, we concluded that different regions and vary types of questionnaires are the key factors for heterogeneity of the above results. Using the same questionnaire to compare the relevant scores of healthy children and children with autism may be more conducive to assessing the difference in GI symptoms prevalence.

The objectivity and reliability of the data may need to be based on the collection of questionnaires from different ASD populations to reduce the spatial bias; the included literature involves reports of parents as well as self-reported data. In addition, children with ASD generally have heterogeneous neurodevelopmental disorders and thus, differences in their abilities to perceive and talk about the severity of individual GI symptoms. Therefore, a simple GI questionnaire may not be sufficient to reflect the actual situation, and it needs to be supplemented with neurodevelopmental, psychological, behavioral, stool sample, and other evaluations (43). The potential influence of GI symptoms on the severity and progression of ASD has always been concerning. Therefore, a comprehensive evaluation tool for the GI symptom scale needs to be further improved and upgraded.

The review showed that children with autism were more likely to have gastrointestinal symptoms than healthy children. The prevalence rate was 55%, versus 26% in neurotypical children. Thus, among children with gastrointestinal symptoms, the prevalence rate of ASD is approximately twice that of TD children. Sharp mentioned that children with autism are more likely than TD children to have eating issues such as food selectivity, food refusal, and poor oral intake, which indicates that children with autism have feeding problems (44, 45). An important way in which dietary habits may affect behavior or gastrointestinal function is the ability to change the intestinal microbiome. In preclinical and clinical studies, diet rapidly changes the composition of the intestinal microbiota, and the specific microbiota environment is related to changes in behavior, emotion, cognition, and gastrointestinal problems (46, 47). The relationship between breastfeeding and ASD is still controversial. If breastfeeding lasts for 6 months, the risk of autism is lower in some studies ‘report; also the proportion of Clostridium difficile in the intestines of formula-fed infants is higher (48, 49). Some studies have also suggested that ASD is not associated with a history of breastfeeding. However, there was a significant positive relationship between early breastfeeding initiation and less severe core symptoms of autism on Childhood Autism Rating Scale scores (*U* = 405, *p* = 0.017) and better intellectual functions on intelligence quotient score (*U* = 18, *p* = 0.03) (50, 51) Increased microflora and decreased microbial diversity are characteristics of the ASD intestinal microbiota (52, 53).

At the same time, we found that the prevalence of GI in ASD varies among different regions. In the included literature, the prevalence of GI was the highest in Europe, followed by the Americas and Asia. There were few relevant literatures in Africa, and only one paper was included in this study, so it was not included in the scope of discussion. This may be related to the difference of intestinal flora among people in different regions. Geographical region and dietary intake have previously been shown to independently influence the microbial communities (54). Rinninella suggested that each human's gut microbiota are shaped in early life, but differ between individuals due to enterotypes, body mass index (BMI) level, exercise frequency, lifestyle, and cultural and dietary habits. The European diet with animal protein and lipids mainly formed enterotype Bacteroides, while the African or Asian diet with millet/sorghum and local vegetables principally formed enterotype Prevotella. Shifts in plant-based and animal-based diets will change firmicutes levels quickly and repeatably (55, 56). In a review of children's gut microbiota composition, it was noted that African (31.6%) and Central American children (35.7%) had lower percentages of firmicutes. There were significant differences between firmicutes and Bacteroides in European and Central American children. In European and North American children, firmicutes to Bacteroides ratios were higher than those in African and Central American children, and Asian children were in the between. Asian children have significantly higher rates of actinomycetes than Central American children (54). Studies on the composition of the gut microbiome in populations with ASD comorbid GI symptoms vary, with most suggesting that ASD patients exhibit decreased Bacteroides, elevated Firmicute phylum levels, low levels of enterococcus and Bifidobacteria, and higher abundance of Actinomycete and beta Proteobacteria (57). Iglesias-Vázquez et al. conducted a meta-analysis of intestinal microbial composition in children with ASD, and showed that children with ASD showed a significantly higher abundance of the genera Bacteroides, Parabacteroides, Clostridium, Faecalibacterium, and Phascolarctobacterium and a lower percentage of Coprococcus and Bifidobacterium. By analyzing the data provided in the literature, we found that the abundance of corresponding bacteria genera in the literature reports of different regions was significantly different. The Bacteroides’levels in In children with ASD in Asia (31.44%) was significantly higher than that in America (20.47%) and Europe (9.05%), while the Clostridium in Europe (6.24%) was higher than that in America (0.05%) and Asia (0.65%), and the Bifidobacterium’levels was higher in Europe (7.75%) and Asia (5.87%). It was lowest in the Americas (0.37%) (58). The diet, living habits and consumption level in different regions have a huge impact on the intestinal microbes of people in the region, which may be one of the reasons for the differences in the prevalence of GI symptoms in children with ASD in different regions.

The prevalence rate of gastrointestinal symptoms in individuals diagnosed with ASD is two times higher than that of typical children, possibly due to feeding methods and autoimmune. The reason for the increases in the IgG subclasses, IgG2 and IgG4, in the serum of patients with certain autism remains unclear. Serum levels of *γ* immunoglobulins are increased in autoimmune diseases. Hypergammaglobulinemia, related to autoimmunity, can be partly attributed to the activation of polyclonal B cells. Therefore, patients with autism exhibit elevated levels of autoimmune changes. The concentrations of IgG2 and IgG4 in the serum of patients with inflammatory bowel disease also increase. At the same time, children with autism are often accompanied by inflammatory bowel-like intestinal symptoms such as diarrhea, constipation, flatulence, and esophageal reflux (59). Children with autism often have food selectivity and inadequate nutrient intake. Different dietary structure affects the composition of intestinal microbes, which in turn causes gastrointestinal problems (42).

We found that the most common GI symptom in children with ASD was constipation, followed by food intolerance, and the prevalence of abdominal distension and pain was more than 20%. A cross-sectional study by Harris (60) found that ASD in children was positively correlated with constipation, and the indirect effects of food selectivity (pickiness) on both of these cannot be ignored. In addition, there are multidirectional and complex interactions between changes in intestinal microbial function, ASD, and constipation. The low content of Bacteroides, Firmicutes, and six other diet-related bacteria may increase the prevalence of constipation in ASD patients (61).

Constipation is also significantly related to the toilet resistance of In children with ASD. At the same time, it is related to delayed language expression and low social motivation. However, these can be improved through toilet training and other social skills training (62). As In children with ASD show more difficulties completing the above training than typical children, the vicious cycle of constipation and toilet resistance makes In children with ASD even more prone to constipation. There is a significant two-way relationship between gastrointestinal symptoms and internalization symptoms such as anxiety and sleep disorders in children with ASD (63). Lower GI symptoms, such as constipation, correlate significantly with decreases in parasympathetic tone (64). Anxiety caused by parasympathetic activity at baseline is closely associated with lower gastrointestinal symptoms (65). It can be inferred that the probability of anxiety in children with ASD is also significantly higher than that in TD children. The prevalence of constipation in ASD is positively correlated with anxiety severity and age. The likelihood of constipation among older children with increased anxiety increased by 11% (66). A case-control study on gastrointestinal function and anxiety in autistic children showed that the anxiety of In children with ASD may be partly explained by the presence of gastrointestinal symptoms, and that the occurrence of abdominal pain and vomiting in children with ASD may be more related to anxiety (30).

Autism is often accompanied by autoimmune abnormalities. A large-sample, multi-ethnic, and national cross-sectional study showed that allergic diseases, especially food allergy, are more closely related to autism, and their mechanism may be related to the gut brain behavior axis, involving changes in the gut microbiome, allergic immune activation, neurodevelopmental abnormalities, and other biological processes (67). A national health survey report from 2011 to 2015 showed that the prevalence of food allergy reported by the parents of autistic children (13.1%) was nearly 2.5 times that of non-autistic children (5.4%) (68). A meta-analysis showed that food hypersensitivity was positively associated with ASD, whereas girls and children under 12 years of age were more likely to present this association (69). Another report gives a contrasting conclusion that young men with autism are more likely to develop food allergy, indicating that sex is an uncertain factor. Sex hormones affect the number of T lymphocytes, and immune-related genes show characteristics of immune dimorphism (70). In addition, food allergy in ASD (especiallyβ-lactoglobulin sensitization, the main allergen in milk) is closely related to the mechanism of “brain allergy,” which includes the loss of the intestinal barrier, imbalance of intestinal flora, abnormal morphology of astrocytes, and brain TNF-α-related inflammation. This also increases the risk of anxiety and depression in autism (71).

The symptoms of GI tract involvement such as constipation, food allergy, abdominal pain, abdominal distension, and diarrhea are mostly considered to be related to the metabolic disorder of “microbiota gut brain axis” (73). Their severity is closely related to the repetitive and stereotypical behavior of ASD, but not significantly related to social and communication difficulties (40). At the same time, they are also related to increased self-mutilation (such as arm biting, head banging, and hair pulling, that occur without an apparent intent of willful self-harm but may pose significant risk of harm to self) (59), aggressive behavior, sleep disorders, and attention deficits (23); therefore, improving the symptoms of ASD by treating gastrointestinal symptoms would be a good idea. Clinicians and parents should take the high prevalence of gastrointestinal symptoms in patients with autism very seriously.

## 8. Limitations and prospects

This study mainly contributes to estimating the prevalence of GI symptoms among those children with autism, as well as adding to the evidence of its high prevalence. However, there is also a defect in this study where the factors leading to heterogeneity, such as children's age, race, family income, parent's education level, and mother's childbearing age, etc., are unfortunately not able to be considered and further discussed because of the limited data within the references included.

## 9. Conclusion

The results of this meta-analysis on GI symptoms related to ASD demonstrate that children with ASD are more prone to develop GI symptoms than TD children, in spite of the data discrepancy in its prevalence reported in the literature, which may be accounted for, in a way, by the variety in respondents of surveys conducted in different regions. Another hard nut to crack in this study is that, the deviation of expression of children with ASD, the difficulty in communication with them, and the varieties of questionnaires that have been used, can all be the factors leading to an unsatisfactory scenario where the current mainstream GI symptom scale may not comprehensively reflect the true GI symptoms of children with ASD. In conclusion, this study does provide a foundation for further researches in such fields, as well as contributing to better comprehension and treatment towards gastrointestinal symptoms in individuals diagnosed with AsD, but still has limitations remained to be overcome in the future.

## Data Availability

The original contributions presented in the study are included in the article/**Supplementary Material**, further inquiries can be directed to the corresponding author.

## Supplementary material

SUPPLEMENTARY FIGURE S1

The total prevalence of GI in children in TD group. Removing the articles that differed significantly from the others, the prevalence was 19%, *I*^2^ = 83.49%, *P* <0.05.

SUPPLEMENTARY FIGURE S2

Constipation in children with ASD and TD, with an OR value of 2.83, *I*^2^ = 23.6%, *p* > 0.05. Fixed effects model.

SUPPLEMENTARY FIGURE S3

Seven studies compared abdominal pain in ASD and corresponding TD controls, with an OR of 2.60 (95% CI: 2.07, 3.26). Fixed effects model.

SUPPLEMENTARY FIGURE S4

The prevalence of diarrhea in ASD and TD groups. The combined OR was 3.27 (95% CI: 1.81, 5.91). Random effects model.

SUPPLEMENTARY FIGURE S5

The prevalence of abdominal distension in ASD and TD. The OR was 1.45 (95% CI: 0.96, 2.17). Fixed effects model.

SUPPLEMENTARY FIGURE S6

The prevalence of vomiting in ASD and TD groups. OR of 3.18 (95% CI: 2.12, 4.77). Fixed effects model.

SUPPLEMENTARY FIGURE S7

The cumulative prevalence of various symptoms showed that 37% of the children with ASD had constipation. (95% CI: 0.30, 0.43), *I*^2 ^=^ ^96.00%, *p*^ ^=^ ^0.00. Random effects model.

SUPPLEMENTARY FIGURE S8

According to 22 articles, diarrhea in children with ASD, with a combined prevalence of 19% (95% CI: 0.14, 0.24), *I*^2 ^=^ ^96.86%, *p*^ ^=^ ^0.00. Random effects model.

SUPPLEMENTARY FIGURE S9

Abdominal pain in children with ASD with a prevalence of 21% (95% CI: 0.14, 0.28), *I*^2 ^=^ ^96.55%, *p*^ ^=^ ^0.00. Random effects model.

SUPPLEMENTARY FIGURE S10

Vomiting was collected from 17 articles, with a prevalence of 8% (95%CI: 0.05, 0.12), *I*^2 ^=^ ^94.11%, *p*^ ^=^ ^0.00. Random effects model.

SUPPLEMENTARY FIGURE S11

The prevalence of abdominal distension in the 12 groups of children with ASD was 23% (95% CI: 0.12, 0.37), *I*^2 ^=^ ^97.63%, *p*^ ^=^ ^0.00. Random effects model.
