# Supplementary figures and images for: Questionnaire-based analysis of autism spectrum disorders and gastrointestinal symptoms in children and adolescents: a systematic review and meta-analysis
Source: Front Pediatr. 2023 Jul 26;11:1120728. doi: 10.3389/fped.2023.1120728 (PMC10410855; doi:10.3389/fped.2023.1120728)

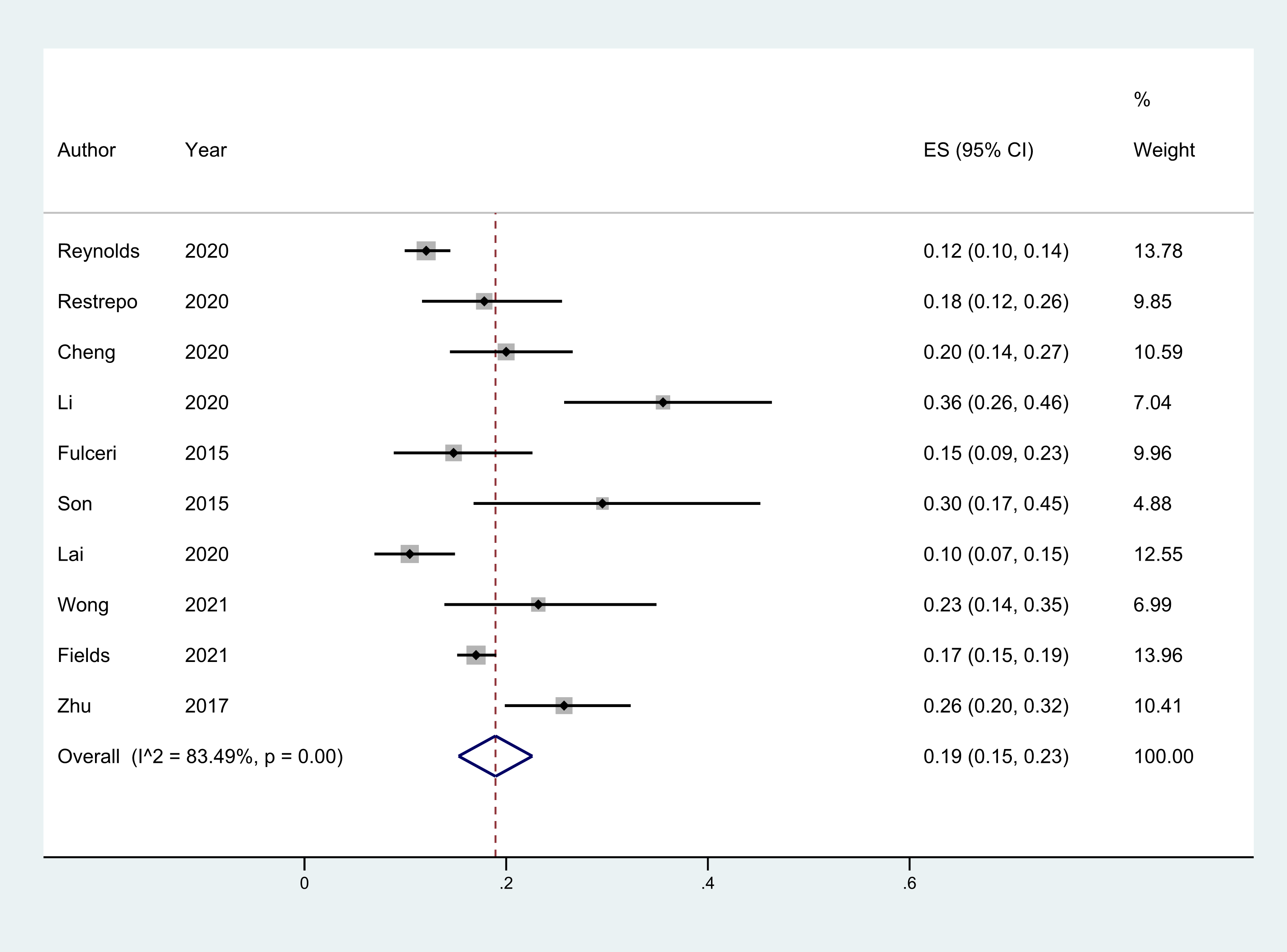

Supplement: Supplementary file 1 [file Datasheet1.zip › Figure 1.tiff]

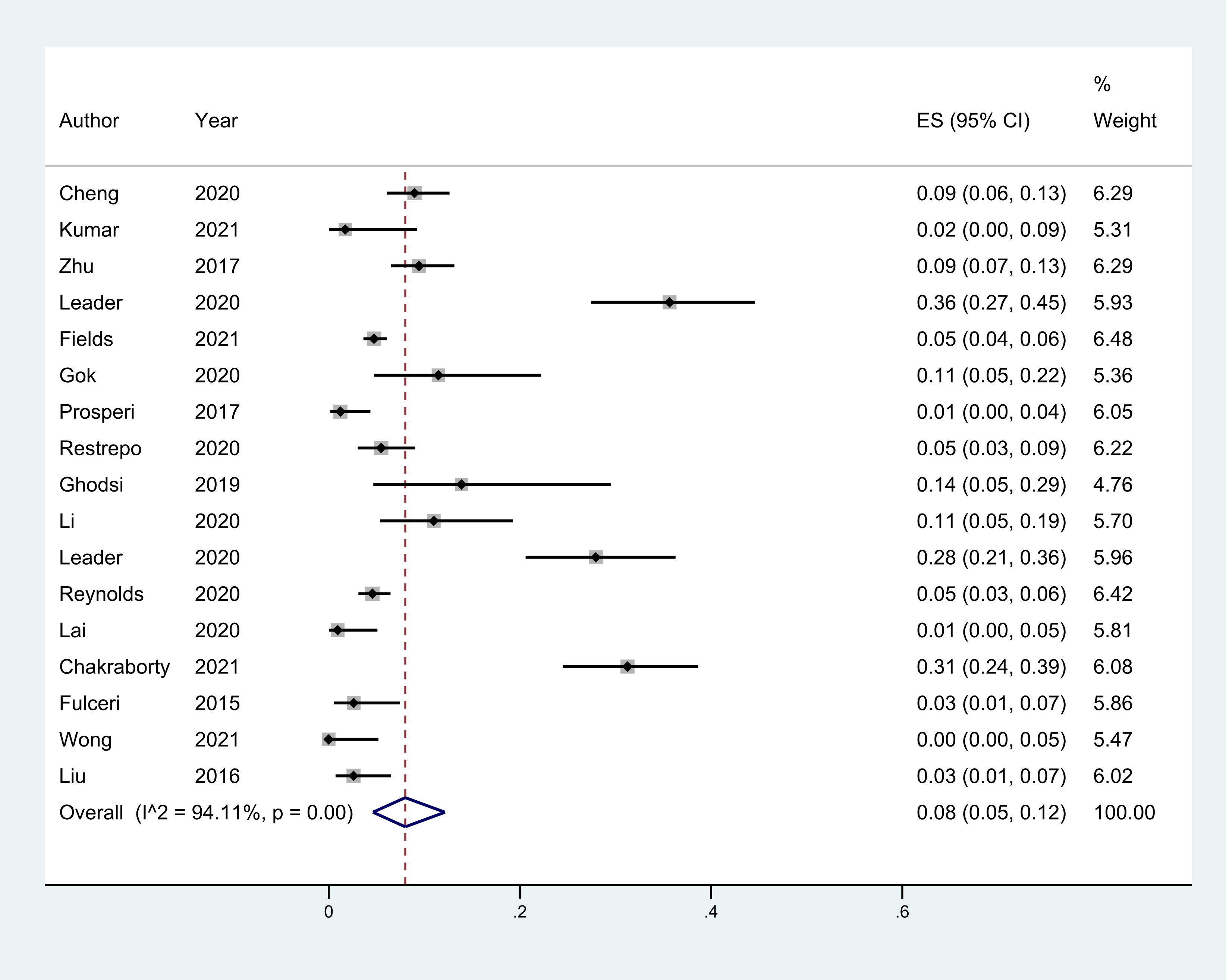

Supplement: Supplementary file 1 [file Datasheet1.zip › Figure 10.tiff]

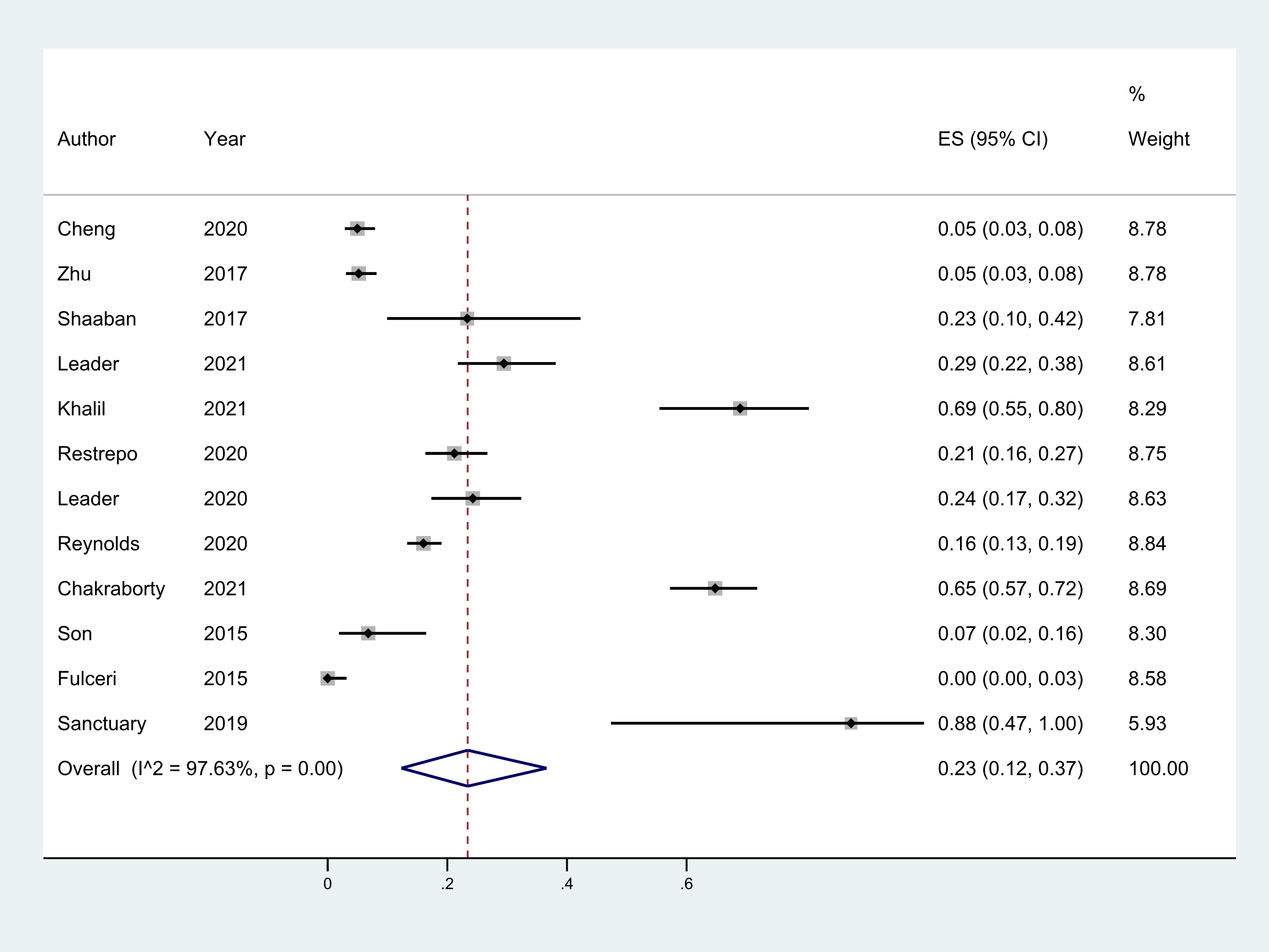

Supplement: Supplementary file 1 [file Datasheet1.zip › Figure 11.tiff]

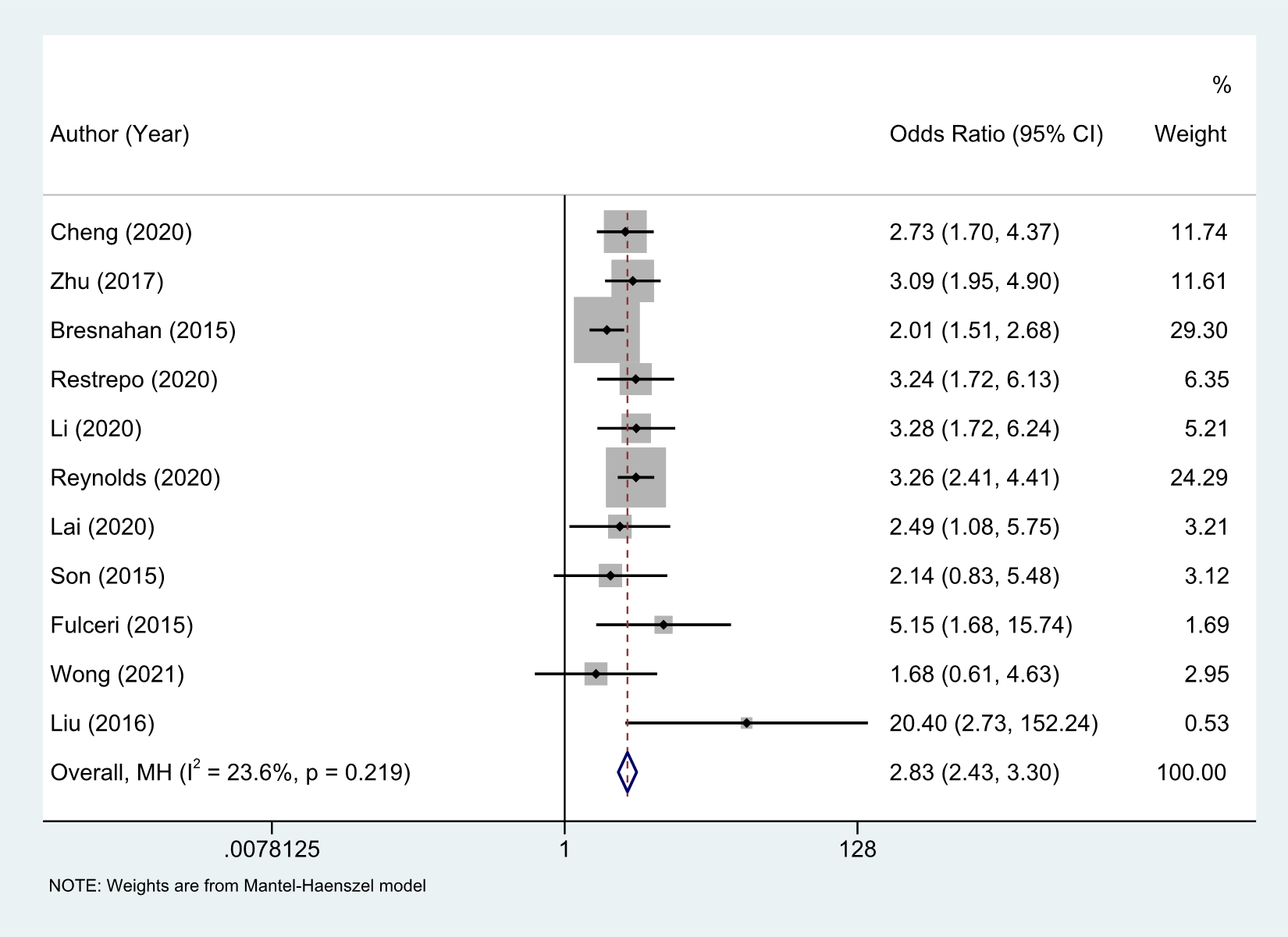

Supplement: Supplementary file 1 [file Datasheet1.zip › Figure 2.tiff]

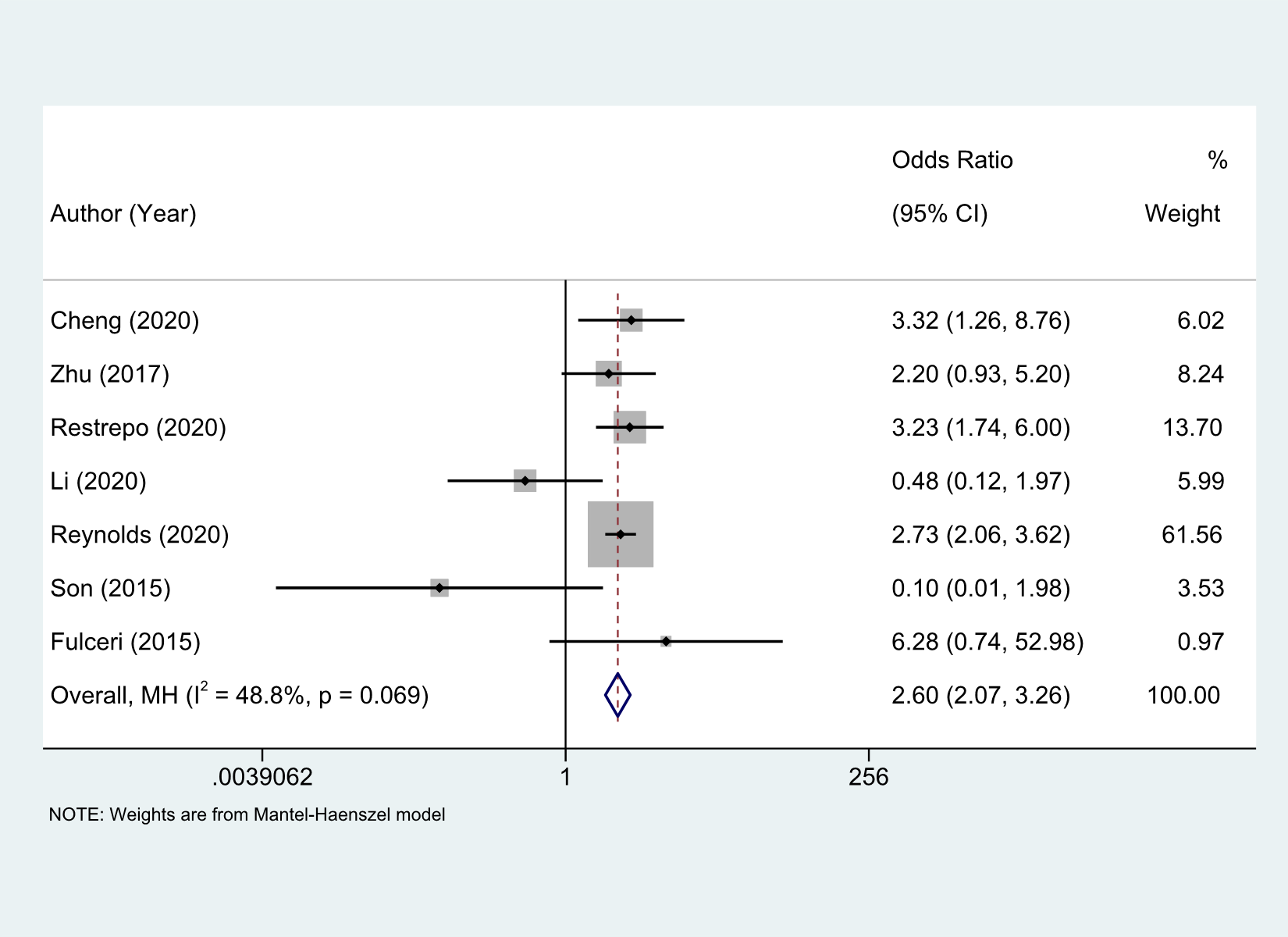

Supplement: Supplementary file 1 [file Datasheet1.zip › Figure 3.tiff]

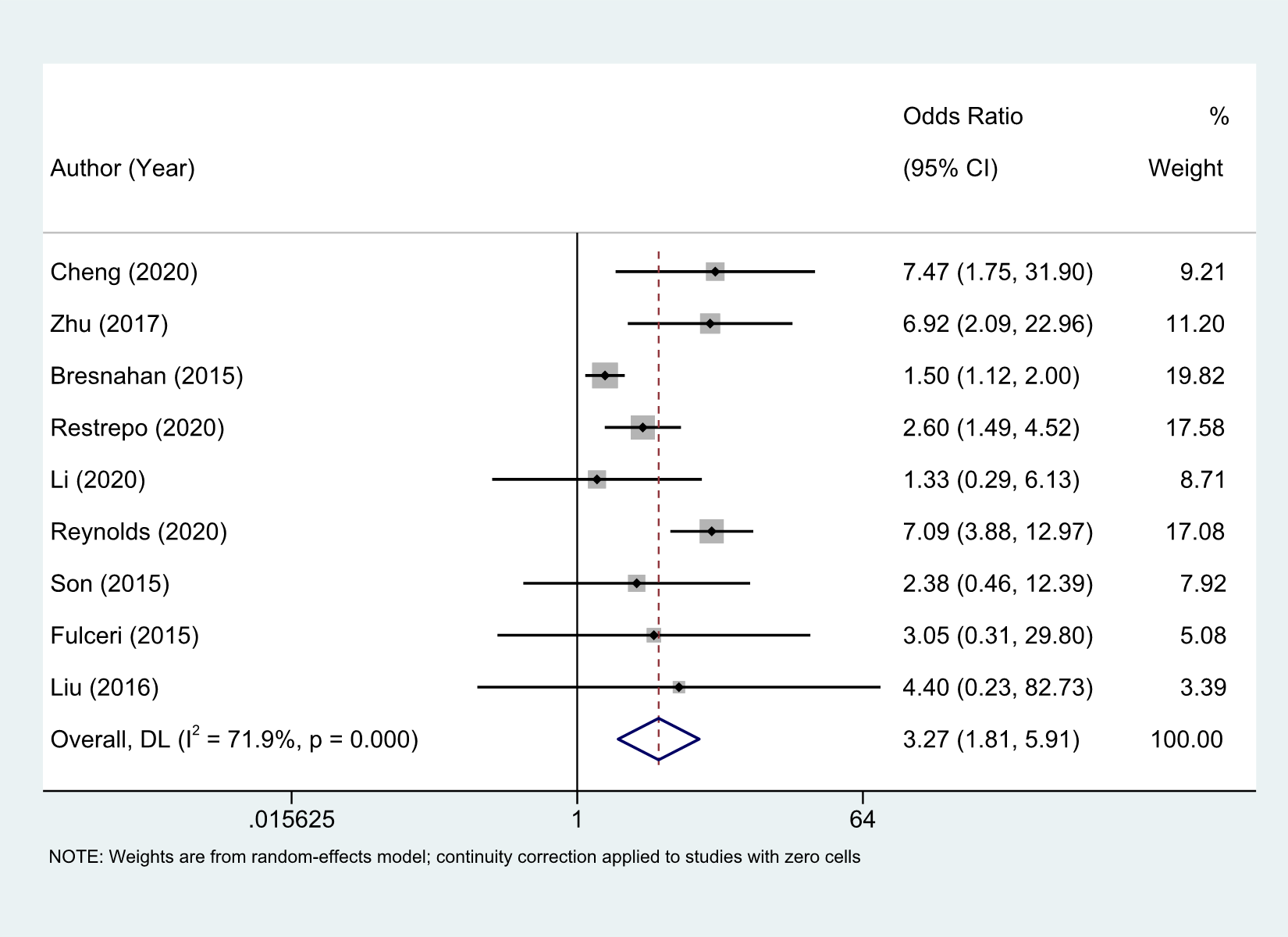

Supplement: Supplementary file 1 [file Datasheet1.zip › Figure 4.tiff]

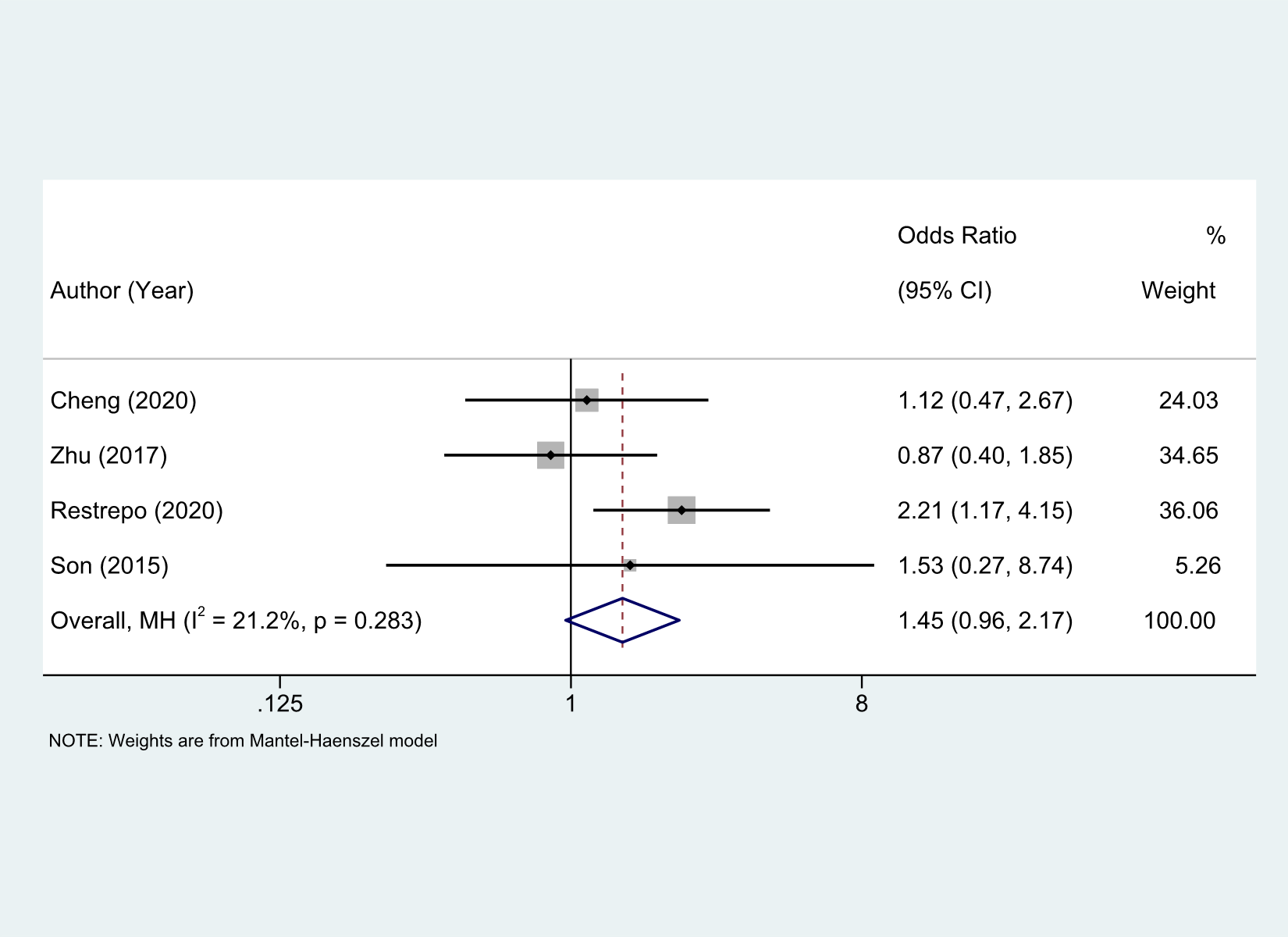

Supplement: Supplementary file 1 [file Datasheet1.zip › Figure 5.tiff]

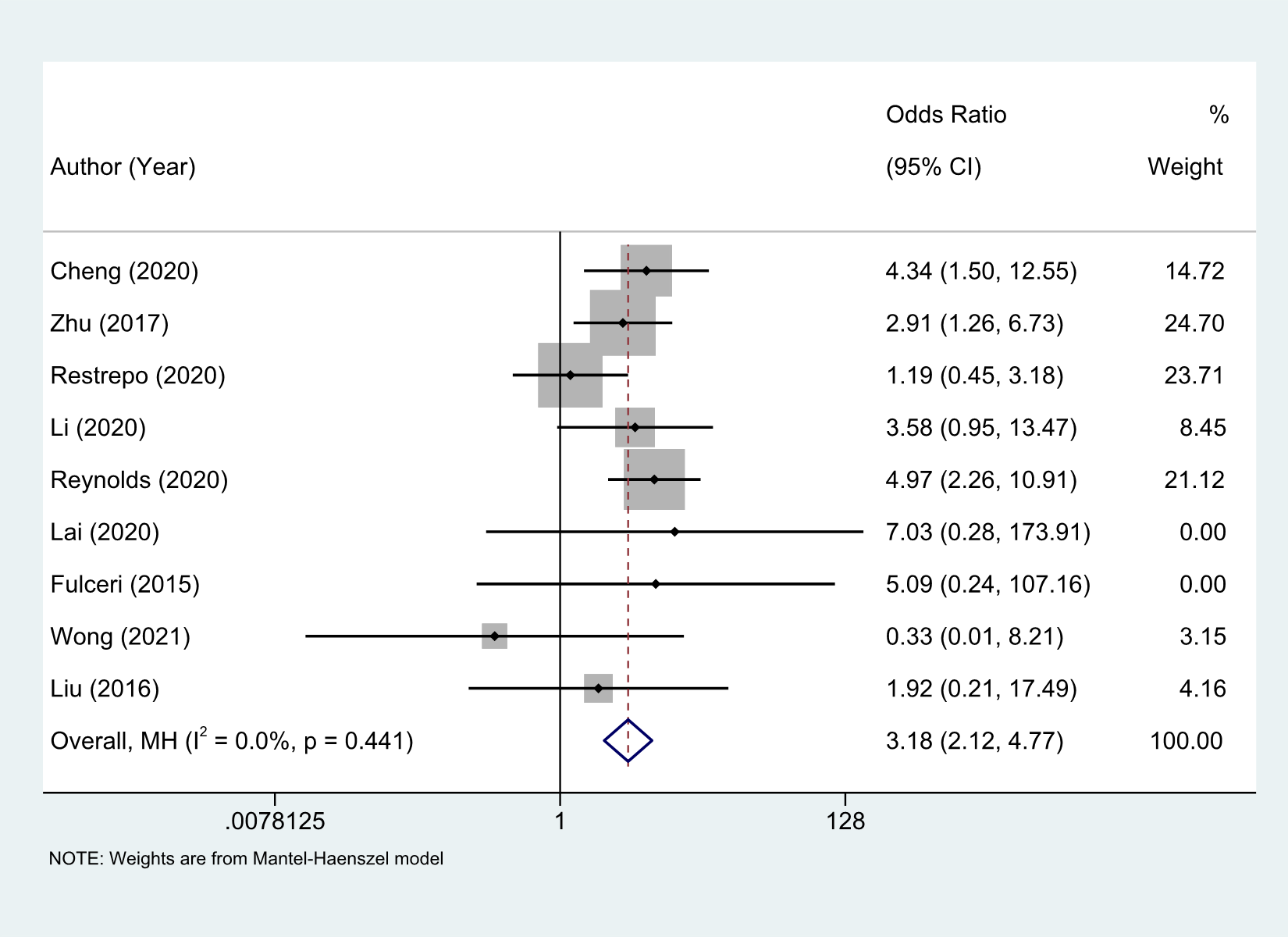

Supplement: Supplementary file 1 [file Datasheet1.zip › Figure 6.tiff]

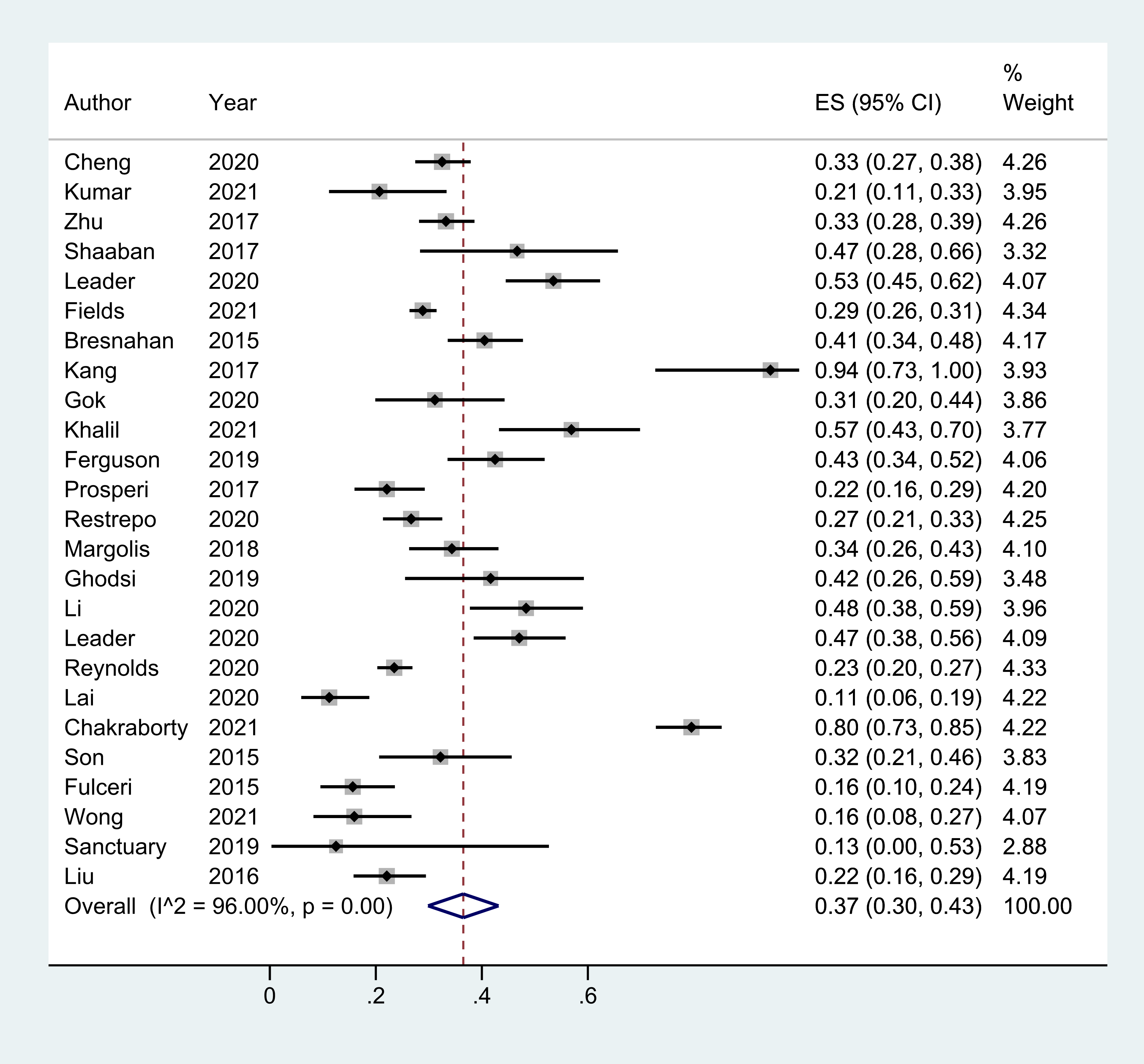

Supplement: Supplementary file 1 [file Datasheet1.zip › Figure 7.tiff]

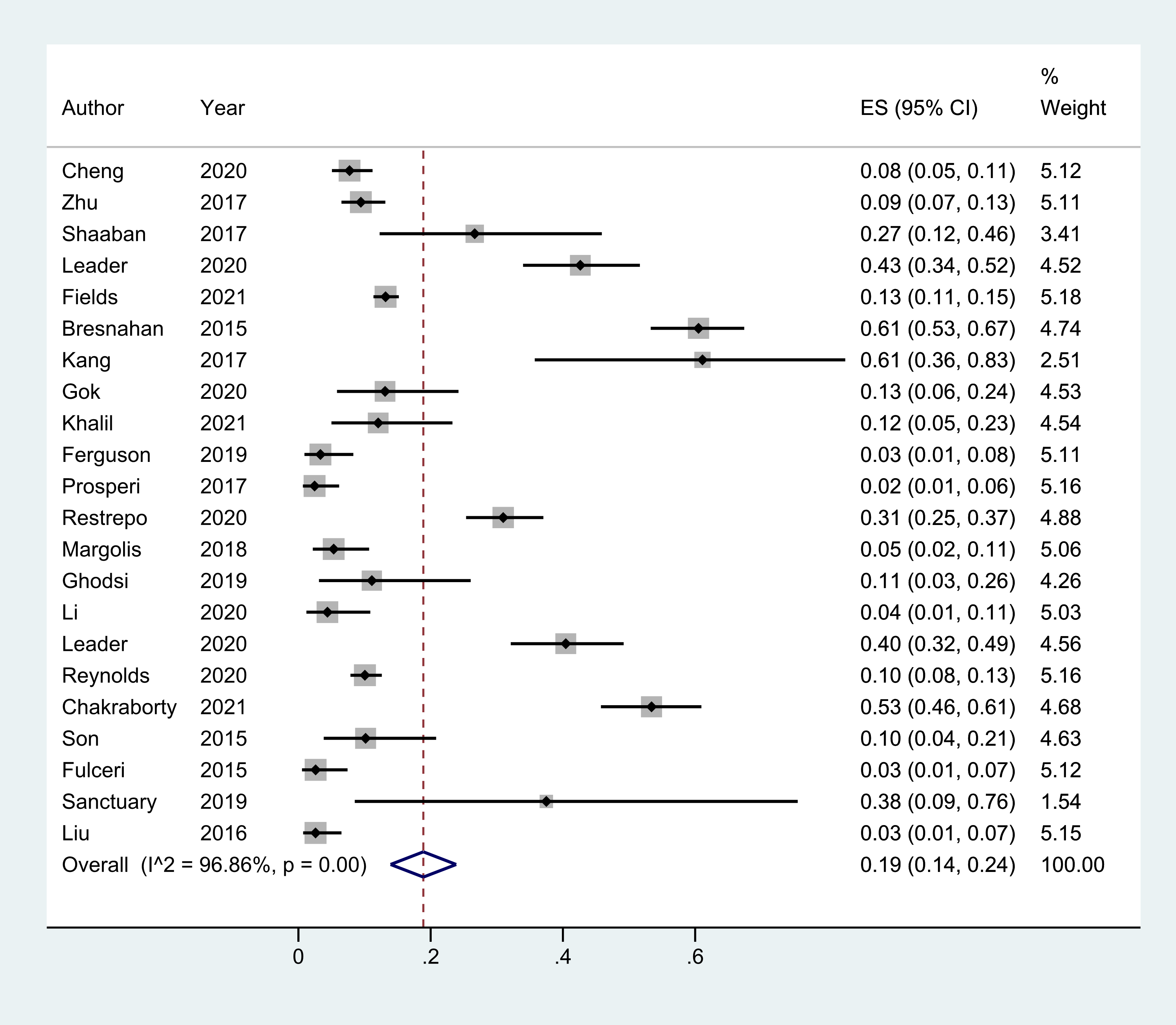

Supplement: Supplementary file 1 [file Datasheet1.zip › Figure 8.tiff]

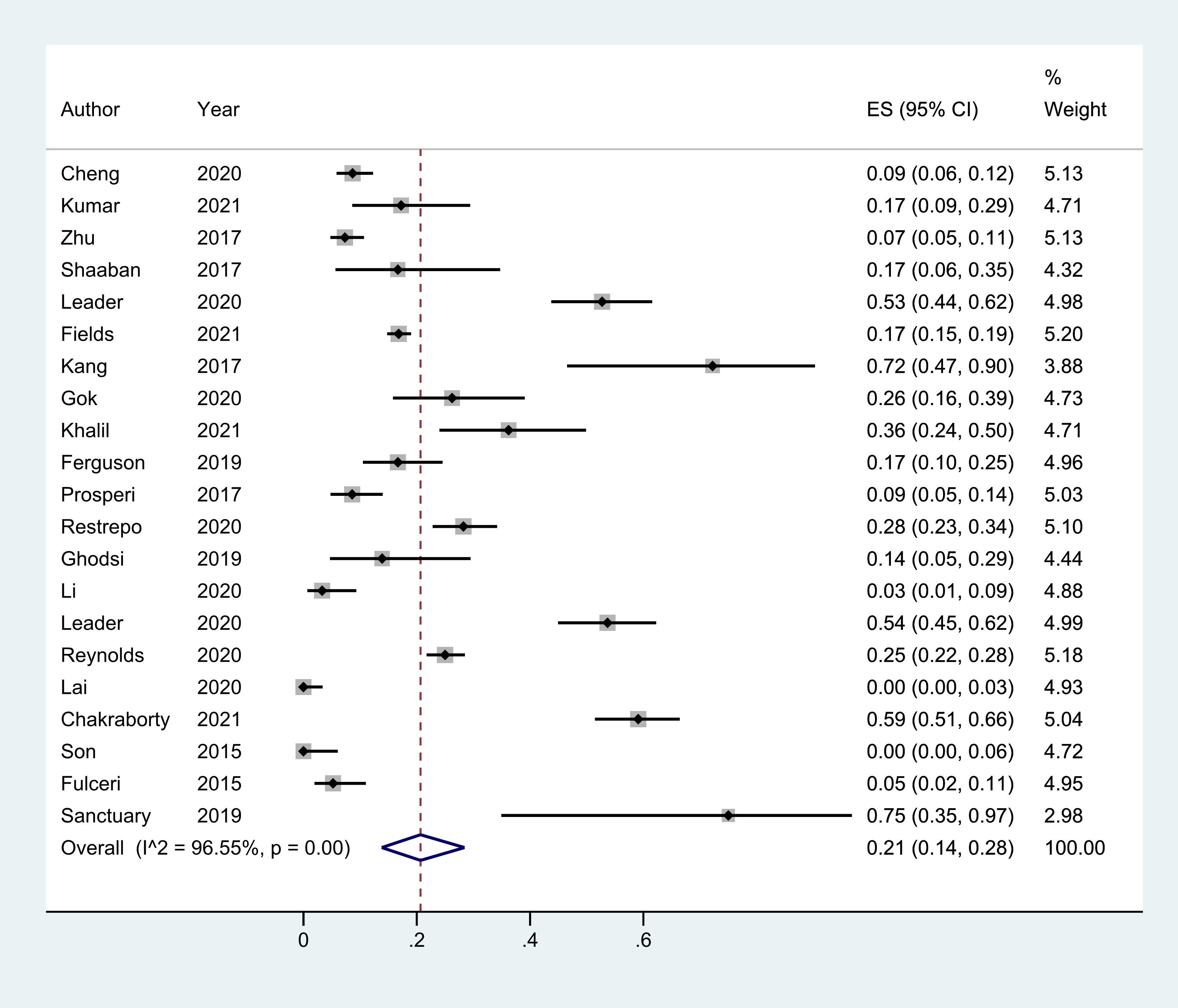

Supplement: Supplementary file 1 [file Datasheet1.zip › Figure 9.tiff]
